# Safety, efficacy, gastrointestinal tolerance, and digestibility of brewed chicken protein in healthy adult dogs

**DOI:** 10.3389/fvets.2025.1593209

**Published:** 2025-07-07

**Authors:** Meredith A. Smola, Patrícia M. Oba, Julio C. Mioto, Pernilla Audibert, Tomas Belloso, Kelly S. Swanson

**Affiliations:** ^1^Department of Animal Sciences, University of Illinois Urbana-Champaign, Urbana, IL, United States; ^2^Bond Pet Foods, Inc., Boulder, CO, United States; ^3^Division of Nutritional Sciences, University of Illinois Urbana-Champaign, Urbana, IL, United States; ^4^Department of Veterinary Clinical Medicine, University of Illinois Urbana-Champaign, Urbana, IL, United States

**Keywords:** canine nutrition, novel protein, pet food, precision fermentation, sustainability

## Abstract

**Introduction:**

Producing enough protein continues to be a challenge, but alternatives may provide economic and ecological relief. Sufficient testing is necessary to confirm safety and evaluate nutritional value. Our objective was to evaluate the safety, efficacy, gastrointestinal tolerance, and apparent total tract digestibility (ATTD) of brewed chicken protein (BCP; *Saccharomyces cerevisiae* expressing a chicken protein).

**Methods:**

Thirty-two healthy adult dogs (BW = 9.68 ± 1.18 kg; age = 4.16 ± 1.85 yr) were used in a completely randomized design (*n* = 8/treatment). After a 2-wk acclimation phase, baseline measurements were collected and dogs were allotted to the following treatments and fed for 26 wk: control diet (0% BCP; Control), 15% BCP (Low), 30% BCP (Medium), or 40% BCP (High). Palatability was assessed by comparing dry diets coated with 0% (control) vs. 1% BCP in 20 adult dogs. Data were analyzed using the Mixed Models procedure of SAS 9.4, with *p* < 0.05 being significant and trends accepted at *p* < 0.10.

**Results:**

Consumption of BCP did not affect food intake, BW, physical parameters, serum chemistry, hematology, and urinalysis. The dry matter, organic matter, and crude protein ATTD were greater (*p* < 0.05) for High, while the fat ATTD was greater (*p* < 0.05) for Control. Fecal output was lower (*p* < 0.0001) and fecal dry matter was lower (*p* < 0.001) for dogs fed High. Fecal acetate concentrations were lower (*p* < 0.05) and propionate concentrations tended to be higher (*p* = 0.06) in dogs fed BCP. Fecal isobutyrate, isovalerate, indole, total phenol and indole, and ammonia concentrations were lower (*p* < 0.001) and fecal valerate concentrations were higher (*p* < 0.0001) in dogs fed BCP. Fecal bacterial alpha diversity was lower (*p* < 0.05) in dogs fed BCP. For beta diversity, dogs fed Control were different than those fed BCP. Over 20 fecal bacterial genera were affected by BCP consumption. Palatability of BCP was high (*p* < 0.05; 2.93:1 consumption ratio).

**Conclusion:**

These results indicate that the BCP ingredient tested is an effective source of protein that is safe for use in adult dog foods at an inclusion level of up to 40%. No detrimental effects were observed, and notable changes to nutrient digestibility and fecal characteristics, metabolites, and microbiota populations suggest potential benefits on gastrointestinal health.

## Introduction

The global human population will grow to around 9.8 billion by 2050 and 10.4 billion by 2,100, according to the United Nations (1). The global pet population is currently estimated at 1 billion (2), with the U. S. dog and cat populations projected to increase from 85 million to over 100 million and from 65 million to 82 million, respectively, by 2030 (3). The anticipated growth in human and pet populations will continue to increase demands for food production, particularly protein-based ingredients. For example, projections indicate that the demand for animal-derived proteins is expected to double by the year 2050 (4), thereby intensifying the pressure on agricultural sectors to generate adequate food supplies. Currently, animal-derived proteins from livestock account for approximately 18% of global calorie consumption and 25% of protein intake (4). However, this sector faces significant environmental challenges, including pasture degradation, soil erosion, loss of biodiversity, disruption of water cycles, and greenhouse gas emissions (5).

Chicken is among the most consumed animal proteins globally and is widely used in companion animal nutrition due to its palatability and amino acid profile. However, conventional chicken production presents several nutritional, economic, and environmental challenges that have been reviewed by Gržinić et al. (6). There are environmental and health challenges associated with intensive poultry farming, which are pertinent to the use of chicken protein in animal diets. Waste materials such as poultry litter and manure can pose significant threats to environmental and human health if not properly managed. Poultry waste may emit ammonia, nitrous oxide, and methane, contributing to global greenhouse gas emissions and may contain other residues and pathogens that may contaminate air, soil, and water if not managed properly. Dust emitted from intensive poultry operations contains various pollutants that can adversely impact the health of poultry, farm workers, and nearby inhabitants. Furthermore, fastidious odors from poultry operations can negatively affect the quality of life of workers and surrounding populations. These realities underscore the need for sustainable practices in poultry farming to mitigate environmental and health risks. Sustainable alternative protein sources that offer environmental relief are imperative for the future of the human and pet food systems.

Yeast (*Saccharomyces cerevisiae*) products are used widely in commercial pet foods, with many being used as a palatant and others serving as functional ingredients that provide beneficial health effects (7–12). Yeast-based ingredients may include components of the yeast cell wall, such as mannanoligosaccharides and *β*-glucans, which improve intestinal health by increasing populations of fecal *Bifidobacterium* and *Lactobacillus*, elevating ileal immunoglobulin (Ig) A concentrations, inhibiting the expression of inflammatory mediators, and enhancing the expression of tight junction proteins that are linked with intestinal permeability (10, 13, 14). Moreover, yeast-based ingredients such as dried brewer’s yeast and grain distillers dried yeast may be used as a significant protein source in pet foods, as they are a rich source of high-quality proteins and amino acids, B vitamins, and minerals (15). As defined by the (16), brewers dried yeast is the dried, non-fermentative, non-extracted yeast of the botanical classification *Saccharomyces* resulting as a by-product from the brewing of beer and ale. It must contain not less than 35% crude protein and be labeled according to its crude protein content. Grain distillers dried yeast is different in terms of processing method, as it is from fermentation of grains and yeast, and separated from the mash, either before or after distillation. It must contain not less than 40% crude protein (16). Yeast as a co-product from ethanol production can be utilized as corn-fermented protein for pet foods as well because it contains over 50% crude protein (17, 18).

Precision fermentation allows for the customization of ingredients containing proteins abundant in the skeletal muscle of animal species. Specifically, DNA from a target animal may be introduced into *S. cerevisiae*, with the fermentation process producing an ingredient composed of both yeast and target animal proteins. Of the complete biomass, the target animal protein typically makes up 10–15% (19). Brewed chicken protein (BCP) is produced using precision fermentation, where microorganisms such as yeast or fungi are bioengineered to produce animal-identical proteins. These proteins are harvested, purified, and formulated into ingredients for pet and human food systems. The fermentation process allows precise control over amino acid composition, enabling the production of highly digestible proteins with consistent profiles. Compared with conventional sources, brewed proteins have much lower biological variability and contaminant concentrations. Precision fermentation dramatically reduces environmental impact by requiring significantly less water and land, producing fewer greenhouse gas emissions, and eliminating the need for animal husbandry and slaughter (20). Brewed chicken protein addresses the shortcomings of conventional sources by offering a reliable supply chain that is independent of animal agriculture, enhancing feed formulation precision with standardized ingredients, supporting environmental, social, and governance goals, and reducing exposure to supply disruptions linked with disease outbreaks (e.g., avian influenza).

Despite their potential benefits, these crafted yeast-based ingredients that are produced by introducing synthesized animal DNA into the *S. cerevisiae* genome have yet to be approved for use in pet foods. Recently, a 6-month feeding study was conducted to evaluate the safety, efficacy, gastrointestinal tolerance, and digestibility of a brewed lamb protein produced using precision fermentation in adult dogs (19). That study demonstrated it is safe to use up to 40% inclusion in adult dogs, but due to their novelty, similar proteins also need to be assessed for their individual safety and efficacy until enough conclusive data for the overall technology is developed. Therefore, the objective of the current study was to assess the safety, efficacy, and apparent total tract digestibility (ATTD) of extruded canine diets containing 0% (Control), 15% (Low), 30% (Medium), or 40% (High) of BCP (*S. cerevisiae* expressing a chicken protein) and to evaluate its effects on the serum chemistry, hematology, and fecal characteristics, metabolites, and microbiota populations of adult dogs. It was hypothesized that all dogs would tolerate BCP and remain healthy throughout the study, without changes to serum chemistry, hematology, and ATTD of macronutrients or energy. Because the BCP is rich in soluble fiber, it was also hypothesized that consumption of BCP-containing diets would increase colonic saccharolytic fermentation, resulting in beneficial shifts in fecal metabolites and microbiota.

## Materials and methods

All procedures were approved by the University of Illinois Institutional Animal Care and Use Committee prior to experimentation (IACUC #23038).

### Animals and treatments

All dogs eligible for use were screened (e.g., physical examination by a board-certified veterinarian, urinalysis, serum chemistry, hematology) prior to the start of the study to confirm health. Thirty-two healthy adult beagle dogs [20 spayed females; 12 neutered males; mean body weight (BW) = 9.68 ± 1.18 kg; mean age = 4.16 ± 1.85 yr] were used in this study. All dogs were housed individually in cages (1.2 m wide × 2.4 m long) in an environmentally-controlled facility at the University of Illinois Urbana-Champaign. Dogs had free access to fresh water at all times. On the basis of the maintenance energy requirement for adult dogs and information from previous feeding records, an amount of food to maintain BW was offered and intake was measured once daily (8–9 am). Dogs had access to an indigestible toy at all times and had other toys, further enrichment, and socialization with each other and humans regularly.

To develop the BCP tested in this study, a comparative bioinformatics system was first used to identify highly abundant proteins in specific animal tissues (e.g., muscle) and then cross-referencing that protein to its corresponding DNA sequence. The DNA sequence from the animal was then codon-optimized for optimal expression in *S. cerevisiae* (strain CEN. PK113-7D) and synthesized by Integrated DNA Technologies (Coralville, IA). The synthesized sequence was placed between native *S. cerevisiae* regulatory elements (promotors and terminators), amplified by polymerase chain reaction, and introduced into a specific site in the *S. cerevisiae* genome. *S. cerevisiae* was then utilized to express the chicken protein under precision fed-batch fermentation conditions at approximately 30°C and a pH of approximately 5.0. The BCP was manufactured in a food-grade facility meeting all regulatory and quality standards established by the U. S Food and Drug Administration, with all raw materials considered safe for use in companion animals. The nutrient solution (primarily dextrose) and filtered air were continuously added during fermentation. Fermentation was complete after all feeding solutions had been added and when the biomass concentration reached approximately 90 g/L on a dry matter (DM) basis. The biomass was then harvested, diluted with water, and underwent centrifugation followed by heat treatment at a minimum of 80°C for 30 min and then spray-dried to produce the dried, inactivated, whole-cell biomass of *S. cerevisiae*. The chicken protein was verified and quantified by liquid chromatography–tandem mass spectrometry (LC–MS/MS) using an Orbitrap Fusion Tribrid mass spectrometer (Thermo Fisher Scientific, Waltham, MA). The final BCP ingredient contains ≥50% crude protein, with ≥10% of crude protein being chicken protein. On an as-is basis, the specific BCP tested in this study (Lot # CLC-231125) contained 53.0% crude protein, 4.8% crude fat, 27.8% total dietary fiber (26.0% insoluble fiber; 1.8% soluble fiber), 5.2% moisture, and 6.3% ash.

All experimental diets were formulated to meet all AAFCO (16) nutrient profiles for adult dogs at maintenance. Diets containing different inclusion levels of BCP [0% (Control), 15% (Low), 30% (Medium), or 40% (High)] were developed (Table 1). The control diet was based on chicken by-product meal (low-ash), brewers rice, chicken fat, and dietary fibers (beet pulp; cellulose). The BCP recipes were formulated so that BCP primarily replaced chicken by-product meal (low-ash), brewers rice, and the fiber sources so that all diets would be similar in nutrient content. All four diets were made at Wenger Manufacturing, Inc. (Sabetha, KS) following good manufacturing practice guidelines.

**Table 1:** List of ingredients and analyzed chemical analysis of the experimental diets tested.

Item	Control	Low	Medium	High
Ingredient composition				
Brewers rice	33.92	34.00	27.86	27.30
Chicken by-product meal, low ash	37.47	26.87	16.03	10.00
Brewed chicken protein^1^	0.00	15.00	30.00	40.00
Beet pulp	7.00	4.00	3.90	0.00
Cellulose	6.50	4.56	1.00	0.00
Chicken fat	5.71	6.58	7.18	7.29
Liquid palatant^2^	3.50	3.50	3.50	3.50
Whole corn	2.00	2.56	6.20	7.48
Menhaden oil	1.00	1.00	1.00	1.00
Dicalcium phosphate	1.00	0.00	0.00	0.00
Potassium chloride, 50% K	0.70	0.63	0.63	0.63
Sodium chloride	0.55	0.55	0.55	0.55
Choline chloride	0.40	0.40	0.40	0.40
Mineral premix^3^	0.13	0.13	0.13	0.13
Vitamin premix^4^	0.12	0.12	0.12	0.12
Limestone	0.00	0.10	1.50	1.60
Analyzed composition				
Dry matter, %	91.82	92.25	92.32	91.33
------ Dry matter basis ------				
Organic matter, %	92.50	93.43	92.58	93.21
Ash, %	7.50	6.57	7.42	6.79
Crude protein, %	33.47	32.05	32.27	32.16
Acid-hydrolyzed fat, %	12.27	12.51	13.30	12.46
Total dietary fiber, %	17.82	14.82	15.32	15.16
Insoluble fiber, %	13.34	7.87	8.28	4.55
Soluble fiber, %	4.48	6.95	7.05	10.62
Nitrogen-free extract^5^, %	28.94	34.05	31.69	33.43
Gross energy^6^, kcal/kg	4.99	5.00	4.95	4.99
Metabolizable energy^7^, kcal/kg	3.23	3.38	3.37	3.36

^1^Brewed chicken protein (Bond Pet Foods, Inc., Boulder, CO).

^2^Liquid hydrolyzed pork liver palatability enhancer (C15057-Doberman, AFB International, St. Charles, MO).

^3^Provided per kg diet: 13.0 mg Cu (as CuCO_3_), 1.3 mg I (as KIO_3_), 97.5 mg Fe (as C_6_H_5_FeO_7_), 13.0 mg Mn (as MnCO_3_), 0.285 mg Se (as Na_2_SeO_3_), 130.0 mg Zn (as ZnCO_3_), 0.0027 mg Co (as CoSO_4_).

^4^Provided per kg diet: 12,000 IU vitamin A acetate; 1,800 IU vitamin D_3_; 96 IU vitamin E acetate; 1.44 mg menadione sodium bisulfite; 0.072 mg biotin; 0.097 mg cyanocobalamin; 0.72 folic acid; 82.8 mg nicotinic acid; 36.48 mg calcium pantothenate; 20.4 mg pyridoxine-HCl; 20.4 mg riboflavin; 20.4 mg thiamin HCl.

^5^Nitrogen-free extract = 100 – (ash + crude protein + acid hydrolyzed fat + total dietary fiber).

^6^Measured by bomb calorimetry.

^7^Metabolizable energy estimated using modified Atwater factors = (3.5 × crude protein) + (8.5 × crude fat) + (3.5 × nitrogen-free extract).

### Experimental design and timeline

This study used a completely randomized design. The total duration of the study was 28 wk. The study began with a 2-wk acclimation phase, whereby all dogs ate the Control diet. After the acclimation phase, a physical examination was conducted by a board-certified veterinarian, an overnight (at least 12 h) fasted blood sample was collected for measurement of serum chemistry, hematology, inflammatory cytokine [interleukin (IL)-6; tumor necrosis factor (TNF)-alpha] concentrations, and IgE concentrations, a free-catch urine sample was collected for urinalysis, and a fresh fecal sample (within 15 min of defecation) was collected for general characteristics (scores; pH; DM %), IgA concentrations, and microbiota populations.

After the acclimation phase, dogs were allocated to four individual groups of 8 dogs (3 males; 5 females). Each group was then randomly assigned to one of four dietary groups and fed for 26 wk. This number of animals is considered adequate to provide a reliable assessment of safety, tolerance, and utility of the test ingredient in dogs AAFCO (16). All dogs were fed to maintain BW throughout the entire study. This was done to avoid any potential bias due to weight gain, which may occur with ad libitum feeding. Body weight and body condition scoring were measured at the beginning and end of the study and weekly during the study (prior to feeding). Food offered and refusals were measured daily to calculate intake. Measures of gastrointestinal intolerance (e.g., emesis, diarrhea), poor physical appearance, and abnormal behavior were monitored and recorded daily throughout the study.

After treatments had been administered for 28 d, all fecal samples over a 5-d period were collected and used to measure ATTD of macronutrients and energy. During the fecal collection period, a fresh fecal sample (within 15 min of defecation) was collected for measurement of fecal characteristics, IgA concentrations, fermentative metabolite concentrations, and microbiota populations. An overnight (at least 12 h) fasted blood sample was collected at this time so that serum chemistry, hematology, inflammatory cytokine concentrations, and IgE concentrations could be measured. Lastly, a free-catch urine sample was collected for urinalysis.

After treatments had been administered for 26 wk, an overnight (at least 12 h) fasted blood sample was collected for measurement of serum chemistry, hematology, inflammatory cytokine concentrations, and IgE concentrations. A free-catch urine sample was again collected for urinalysis. A fresh fecal sample (within 15 min of defecation) was collected for measurements of fecal characteristics, IgA concentrations, and microbiota populations. On the last day of the study, a physical examination was conducted by a board-certified veterinarian.

### Fecal collection

During fecal collections, dogs were housed in their cages as normal. During the total fecal collection, dogs were checked at least 3 times a day for sample collection. All samples were collected, weighed, and scored according to the following scale: 1 = hard, dry pellets, small hard mass; 2 = hard, formed, dry stool; remains firm and soft; 3 = soft, formed, and moist stool, retains shape; 4 = soft, unformed stool, assumes shape of container; and 5 = watery, liquid that can be poured. Samples were then frozen at-20°C until nutrient analysis. At the end of the collection period, the total feces from each animal were weighed, dried, and ground prior to laboratory analysis.

During the fresh fecal sample collections, fecal pH was measured immediately using an AP10 pH meter (Denver Instrument, Bohemia, NY) equipped with a Beckman Electrode (Beckman Instruments Inc., Fullerton, CA). Fecal aliquots for analysis of phenols and indoles were frozen at −20°C immediately after collection. One aliquot was collected and placed in 2 N hydrochloric acid for ammonia, short-chain fatty acid (SCFA), and branched-chain fatty acid (BCFA) analyses. Aliquots for IgA and microbial analysis were transferred to sterile cryogenic vials (Nalgene, Rochester, NY), frozen immediately on dry ice, and stored at -80°C until analysis. One aliquot was collected for DM determination and conducted according to AOAC (21) using a 105°C oven.

### Urine collection

During urine sample collections, a fresh sample of urine was collected by free catch from each dog while they were housed in their regular cage. Once the samples were collected, they were immediately transferred to sterile cryogenic vials (Nalgene, Rochester, NY) and transported to the University of Illinois Veterinary Medicine Diagnostics Laboratory for urinalysis assessment.

### Blood collection

On blood collection days, a fasted blood sample (at least 12 h overnight) was collected from each dog for measurement of serum chemistry, hematology, inflammatory cytokines (TNF-alpha; IL-6), and IgE. Blood samples were collected via cephalic or jugular venipuncture. The neck and (or) forelimb were shaved to remove excessive amounts of hair. Prior to collection, 70% alcohol was applied to sterilize the area. Once the blood sample was collected, the needle was removed, and pressure was applied over the venipuncture site for 30 s or until bleeding was no longer present. Samples were immediately transferred to appropriate vacutainer tubes: #367985 BD Vacutainer® glass serum tubes with gel for serum separation (Becton Dickinson, Franklin Lakes, NJ) for serum chemistry profiles, inflammatory cytokines, and IgE; and #367842 BD Vacutainer® Plus plastic whole blood tubes with K_2_EDTA additive (Becton Dickinson, Franklin Lakes, NJ) for hematology.

The blood tubes for serum isolation were centrifuged at 1,300 × g at 4°C for 10 min (Beckman CS-6R centrifuge; Beckman Coulter Inc., Brea, CA). A fresh sample was transported to the University of Illinois Veterinary Medicine Diagnostics Laboratory for serum chemistry analysis. Aliquots for IL-6, TNF-alpha and IgE were transferred to sterile cryogenic vials (Nalgene, Rochester, NY), frozen immediately on dry ice, and stored at −80°C until analysis. Concentrations of IL-6 (MBS2021058; MyBioSource, San Diego, CA), TNF-alpha (MBS761131; MyBioSource), and IgE (MBS007318; MyBioSource) were measured using commercial canine-specific enzyme-linked immunosorbent assay (ELISA) kits. K_2_EDTA tubes were cooled (but not frozen), with one aliquot being transported to the University of Illinois Veterinary Medicine Diagnostics Laboratory for hematology analyses.

### Chemical analysis and digestibility calculations

Fecal samples used for digestibility analysis were dried at 55°C in a forced-air oven. All dried dietary treatments and feces were ground in a Wiley Mill (model 4, Thomas Scientific, Swedesboro, NJ) through a 2-mm screen. Diet and fecal samples were analyzed for DM and ash according to the AOAC ((21); method 934.01 and 942.05), with organic matter calculated. Crude protein of the diets and feces were determined by Leco Nitrogen/Protein Determinator [TruMac N, Corporation, St. Joseph, MI; AOAC (21)] total nitrogen values according to AOAC (21); method 992.15. Total lipid content was determined using acid hydrolysis and extraction methods facilitated by ANKOM Technology equipment (Hydrolysis System, XT15 Extractor, and RD Dryer; Macedon, NY). Dietary total dietary fiber was determined according to Prosky et al. (22). Gross energy of dietary and fecal samples was measured using an oxygen bomb calorimeter (model 6,200; Parr Instruments; Moline, IL). Apparent total tract digestibility of macronutrients and energy were calculated using the equation as follows:


$$\text{ATTD}\;(\%)=\frac{\text{Nutrient intake}\;(\frac{g}{d})-\text{fecal output}\;(\frac{g}{d})}{\text{nutrient intake}\;(\frac{g}{d})}\times 100$$

Fecal SCFA (acetate, propionate, and butyrate) and BCFA (valerate, isovalerate, isobutyrate) concentrations were determined by gas chromatography according to Erwin et al. (23) using a gas chromatograph (Hewlett-Packard 5890A series II, Palo Alto, CA) and a glass column (180 cm × 4 mm i.d.) packed with 10% SP-1200/1% H_3_PO_4_ on 80/100 + mesh Chromosorb WAW (Supelco Inc., Bellefonte, PA). Nitrogen was the carrier with a flow rate of 75 mL/min. Oven, detector, and injector temperatures were 125, 175, and 180°C, respectively. Fecal ammonia concentrations were determined according to the method of Chaney and Marbach (24). Fecal phenol and indole concentrations were determined using gas chromatography according to the methods described by Flickinger et al. (25). Fecal IgA concentrations were measured using a commercial canine-specific ELISA kit (MBS018650; MyBioSource).

### Fecal DNA extraction and MiSeq Illumina sequencing of 16S rRNA gene amplicons

Total DNA from fecal samples was extracted using Mo-Bio PowerSoil kits (MO BIO Laboratories, Inc., Carlsbad, CA). The concentration of extracted DNA was quantified using a Qubit 3.0 Fluorometer (Life Technologies, Grand Island, NY). 16S rRNA gene amplicons were generated using a Fluidigm Access Array (Fluidigm Corporation, South San Francisco, CA) in combination with Roche High Fidelity Fast Start Kit (Roche, Indianapolis, IN). The primers 515F (5′-GTGCCAGCMGCCGCGGTAA-3′) and 806R (5′-GGACTACHVGGGTWTCTAAT- 3′) target a 252 bp-fragment of the V4 region of the 16S rRNA gene that was used for amplification (primers synthesized by IDT Corp., Coralville, IA) according to Caporaso et al. (26). CS1 forward tag and CS2 reverse tag were added according to the Fluidigm protocol. The quality of the amplicons was assessed using a Fragment Analyzer (Advanced Analytics, Ames, IA) to confirm amplicon regions and sizes. A DNA pool was generated by combining equimolar amounts of the amplicons from each sample. The pooled samples were then size selected on a 2% agarose E-gel (Life technologies, Grand Island, NY) and extracted using a Qiagen gel purification kit (Qiagen, Valencia, CA). Cleaned size-selected pooled products were run on an Agilent Bioanalyzer to confirm the appropriate profile and average size. Illumina sequencing was performed on a MiSeq using v3 reagents (Illumina Inc., San Diego, CA) at the Roy J. Carver Biotechnology Center at the University of Illinois.

### Microbial data analysis

Fluidigm tags were removed using the FASTX-Toolkit (version 0.0.14), and sequences were analyzed using QIIME 2, version 2023.7 (27) and DADA2 (version 1.14) (28). High-quality (quality value ≥ 20) sequence data derived from the sequencing process were demultiplexed. Data were then denoised and assembled into amplicon sequence variants (ASV) using DADA2 (28). Taxonomy was assigned using the Naive Bayes classifiers trained on the Silva database (v.138) (29–31). Singletons (ASV that are observed fewer than two times) and ASV that have <0.1% of total observations were discarded. An even sampling depth was used to assess alpha diversity and beta diversity measures. Beta diversity was assessed using weighted and unweighted UniFrac distance (32) measures and presented using principal coordinates analysis plots.

### Statistical analysis

Data were analyzed using the Mixed Models procedure of SAS (SAS Institute, Inc., Cary, NC). Differences among dietary treatments were determined using a Fisher-protected least significant difference with a Tukey adjustment to control for experiment-wise error. Data normality was checked using the univariate procedure and Shapiro–Wilk statistic, with log transformation being used when normal distribution is lacking. If after the logarithmic transformation of the data, the data did not reach normality, the data were analyzed using the npar1way procedure and Wilcoxon statistic. A probability of *p* < 0.05 was accepted as statistically significant, with trends accepted at *p* < 0.10. Where significant differences were identified, data were also analyzed using a linear contrast, with p < 0.05 considered significant.

### Palatability test

Twenty adult dogs were used for a standard 2-d palatability test conducted at Kennelwood Inc. (Champaign, IL). A control dry diet coated with 5% of a mixed fat source was compared with the same diet coated with 5% mixed fat source + 1% BCP. Dogs were offered one bowl per day, each containing 800 g of test diets. Food bowls were presented for 30 min each day, and to prevent left–right bias, the bowl position was reversed on the second day of the test. Total daily consumption and first choice preferences were reported for each dog.

## Results

The analyzed dietary chemical composition of the test diets is provided in Table 1. Diets had similar organic matter, ash, crude protein, and fat concentrations. Dietary fiber concentrations were variable across diets, with total and insoluble fiber concentrations being highest for the Control diet. As dietary BCP inclusion increased, insoluble fiber decreased, and soluble fiber increased. Given the slight differences in protein, fiber, and fat across diets, nitrogen-free extract concentration was lowest in the Control diet and highest in the Low diet. Gross energy and metabolizable energy concentrations were similar across all diets.

Baseline BW, body condition scores, food and caloric intakes, fecal characteristics, and fecal IgA concentrations of dogs are presented in Supplementary Table 1. No statistical differences were observed among groups. Baseline serum chemistry, cytokine concentrations, and IgE concentrations of dogs are presented in Supplementary Table 2, with most measures being within the reference ranges for adult dogs. The serum albumin: globulin ratios were above the reference range for all adult dogs. Serum globulin concentrations were below and Na: K concentrations were above the reference ranges for adult dogs allotted to the Control, Low, and Medium diets. Serum alkaline phosphatase was greater (*p* = 0.0127) in dogs allotted to the High diet than dogs allotted to the Medium diet. Alanine transaminase was greater (*p* = 0.0204) in dogs allotted to the Low diet than dogs allotted to the Control and Medium diets. In addition, serum total protein (*p* = 0.0774), globulin (*p* = 0.0607), P (*p* = 0.0930), K (*p* = 0.0525), and triglyceride (*p* = 0.0557) concentrations, anion gap (*p* = 0.0865), and Na: K ratios (*p* = 0.0817) tended to be different among dogs allotted to treatment groups. Baseline serum cytokine and IgE concentrations were not different among groups (*p* > 0.10).

Baseline hematology measures and urine characteristics of dogs are presented in Supplementary Table 3. All measures were within reference ranges for adult dogs, but reticulocyte count was greater (*p* = 0.0011) in dogs allotted to the High diet than dogs allotted to the other diets. Hematocrit was greater (*p* = 0.0373) in dogs allotted to the High diet than those allotted to the Medium diet. Lastly, monocyte % was greater (*p* = 0.0172) in dogs allotted to the Medium diet than those allotted to the Low diet. Baseline urine characteristics were not different among groups.

Baseline bacterial alpha diversity indices of fecal samples, including observed features, the Shannon Diversity Index, and Faith’s phylogenetic diversity, were not different among groups (Supplementary Figure 1). Similarly, baseline fecal bacterial beta diversity, as represented by principal coordinates analysis plots of unweighted and weighted UniFrac distances of microbial communities, were not different among groups (Supplementary Figure 2). The relative abundances of 10 fecal bacterial genera were different among groups at baseline (Supplementary Table 4). Within the Actinobacteriota phyla, the relative abundance of *Adlercreutzia* tended to be different (*p* = 0.0946) among dogs allotted to treatment groups. Within the Firmicutes phyla, the relative abundance of *Dubosiella* was greater (*p* = 0.0362) in dogs allotted to the Low diet than dogs allotted to the Control diet. The relative abundance of *Ruminococcus torques* was greater (*p* = 0.0268) in dogs allotted to the Control diet than dogs allotted to the Low diet. In addition, the relative abundances of *Allobaculum* (*p* = 0.0769), Eubacteriaceae unclassified (*p* = 0.0876), *Fournierella* (*p* = 0.0675), *Holdemanella* (*p* = 0.0857), *Sellimonas* (*p* = 0.832), *Terrisporobacter* (*p* = 0.0875), and *Turicibacter* (*p* = 0.0888) tended to be different among dogs allotted to treatment groups.

After 4 wk on treatment, BW, body condition scores, and food, caloric, and macronutrient intakes were not affected by treatment (Table 2). However, as-is fecal output (*p <* 0.0001), dry fecal output (*p =* 0.0042), and fecal output/food intake (*p* = 0.0084) were greater in dogs fed the Control and Medium diets than those fed the High diet. All three outcomes had significant linear contrasts, with as-is fecal output (*p <* 0.05), dry fecal output (*p <* 0.01), and fecal output/food intake (*p* < 0.05) decreasing linearly with increasing dietary BCP inclusion. The ATTD of DM (*p* = 0.0128) and organic matter (*p* = 0.0175) were greater for the High diet than the Control and Medium diets. The ATTD of crude protein was greater (*p* = 0.0100) for the High diet than the Medium diet. The ATTD of fat was greater (*p* = 0.0003) for the Control than the High and Medium diets. Lastly, the ATTD of energy tended to be different (*p* = 0.0720) among dietary treatments. The ATTD of DM, organic matter, and fat had significant linear contrasts. The ATTD of DM (*p* < 0.05) and organic matter (*p* < 0.05) were linearly increased, while the ATTD of fat (*p* < 0.0001) was linearly decreased with increasing dietary BCP inclusion.

**Table 2:** Body condition scores, body weight, food and caloric intake, and fecal output of healthy adult dogs after consuming test diets for 4 wk and apparent total tract macronutrient and energy digestibility of test diets.

Item	Control	Low	Medium	High	SEM	*p*-value
Body condition score	5.13	5.94	5.00	5.56	0.41	0.3588
Body weight (BW), kg	9.51	9.08	9.04	9.65	0.50	0.7718
Food intake						
Food, g/d (as-is)	204.62	190.13	195.00	175.62	15.71	0.6255
Dry matter (DM), g/d	187.88	175.37	180.12	160.25	14.47	0.5913
Organic matter, g/d	173.87	164.00	166.63	149.50	0.09	0.5747
Crude protein, g/d	65.00	56.13	62.75	51.63	0.09	0.3849
Fat, g/d	23.05	21.94	23.94	19.99	5.64	0.9092
Food intake (DM, g/d)/BW (kg)	19.23	20.68	20.21	18.20	0.12	0.8037
Caloric intake, kcal/d^1^	587.05	592.29	606.50	563.88	49.46	0.9425
Caloric intake (kcal)/BW (kg)^1^	62.06	69.83	68.08	61.27	0.12	0.8017
Fecal output						
Fecal output, as-is (g/d)	128.88^a^	107.88^ab^	128.00^a^	84.75^b^	0.10	<0.0001
Fecal output, DM (g/d)	40.88^a^	36.25^ab^	38.38^a^	24.88^b^	3.01	0.0042
Fecal output (as-is, g/d)/food intake (DM, g/d)	0.70^a^	0.60^ab^	0.73^a^	0.52^b^	0.04	0.0084
Nutrient and energy digestibility						
DM, %	78.23^b^	79.64^ab^	77.51^b^	84.15^a^	1.44	0.0128
Organic matter, %	81.30^b^	81.78^ab^	80.46^b^	86.11^a^	1.26	0.0175
Crude protein, %	81.68^ab^	83.18^ab^	78.90^b^	86.38^a^	1.46	0.0100
Fat, %	94.19^a^	91.46^ab^	88.23^b^	89.66^b^	0.83	0.0003
Energy, %	82.17	82.31	80.67	85.91	1.38	0.0720

^1^Metabolizable energy estimated using modified Atwater factors = (3.5 × crude protein) + (8.5 × crude fat) + (3.5 × nitrogen-free extract).

^a-d^Means within a row with different superscripts differ (*P* < 0.05).

After 4 wk on treatment, fecal DM % was greater (*p* = 0.0007) in dogs fed the Low diet than those fed the Medium and High diet, and greater (*p* = 0.0007) in dogs fed the Control diet than those fed the High diet (Table 3). A significant linear contrast was also observed, with fecal DM % linearly decreasing (*p* < 0.001) with increasing dietary BCP inclusion. Fecal scores and pH were not affected by treatment. Several fecal metabolite concentrations also differed among dietary treatments at wk 4. Fecal acetate concentrations were greater (*p* = 0.0377) in dogs fed the Control diet than those fed the Low diet. Fecal propionate tended to be affected by treatment group (*p* = 0.0595). Fecal isobutyrate (*p* = 0.0008) and isovalerate (*p* = 0.0020) concentrations were greater in dogs fed the Control diet than those fed the Medium and High diets, and greater (*p* < 0.01) in dogs fed the Low diet than those fed the Medium diets. Fecal valerate concentrations were greater (*p* < 0.0001) in dogs fed the High and Medium diets than those fed the Low and Control diets. Fecal indole, total phenol and indole, and ammonia concentrations were greater (*p* < 0.0001) in dogs fed the Control and Low diets than those fed the High and Medium diets. Significant linear contrasts were noted for fecal isobutyrate, isovalerate, valerate, indole, total phenol and indole, and ammonia concentrations, with valerate linearly increasing (*p* < 0.0001) and all other metabolites linearly decreasing (*p* < 0.001) with increasing dietary BCP inclusion. Fecal butyrate, total SCFA, total BCFA, phenol, and IgA concentrations were not different among dietary treatment groups.

**Table 3:** Fecal characteristics and fecal metabolites (μmol/g DM) of healthy adult dogs after consuming test diets for 4 wk.

Item	Control	Low	Medium	High	SEM	*P-*value
Fecal characteristics						
Fecal score^1^	3.19	3.06	3.31	3.06	0.10	0.2780
Fecal pH	6.81	7.13	6.73	6.50	0.25	0.3856
Fecal DM, %	31.14^ab^	33.39^a^	28.60^bc^	27.15^c^	0.99	0.0007
Fecal metabolites						
Acetate	439.16^a^	292.85^b^	387.45^ab^	342.38^ab^	34.82	0.0377
Propionate	169.98	154.69	234.48	247.59	27.90	0.0595
Butyrate	118.14	135.07	159.21	183.31	39.23	0.6681
Total SCFA^2^	727.29	581.60	781.13	773.29	86.47	0.3476
Isobutyrate	24.44^a^	19.68^ab^	9.78^c^	14.27^bc^	2.32	0.0008
Isovalerate	31.83^a^	28.66^ab^	13.64^c^	17.03^bc^	3.49	0.0020
Valerate	4.03^b^	6.04^b^	15.59^a^	21.65^a^	2.24	<0.0001
Total BCFA^2^	60.30	54.38	39.01	52.94	6.22	0.1226
Phenol	0.30	0.43	0.41	0.36	0.04	0.1483
Indole	2.86^a^	3.24^a^	0.82^b^	1.04^b^	0.25	<0.0001
Total phenols and indoles	3.16^a^	3.66^a^	1.23^b^	1.40^b^	0.26	<0.0001
Ammonia	104.60^a^	100.70^a^	51.60^b^	64.01^b^	7.80	<0.0001
Fecal IgA, mg/g	7.72	6.81	6.85	9.08	1.94	0.2062

^1^Fecal scores: 1 = hard, dry pellets, small hard mass; 2 = hard, formed, dry stool, remains firm and soft; 3 = soft, formed, and moist stool; retains shape; 4 = soft, unformed stool, assumed shape of containers; 5 = watery, liquid that can be poured.

^2^Total short-chain fatty acids (SCFA) = acetate + propionate + butyrate; Total branched-chain fatty acids (BCFA) = valerate + isovalerate + isobutyrate. Valerate is technically a SCFA, but it is derived from amino acid fermentation so it was included in the BCFA calculation.

^a-d^Means within a row with different superscripts differ (*p* < 0.05).

**Figure 1:**
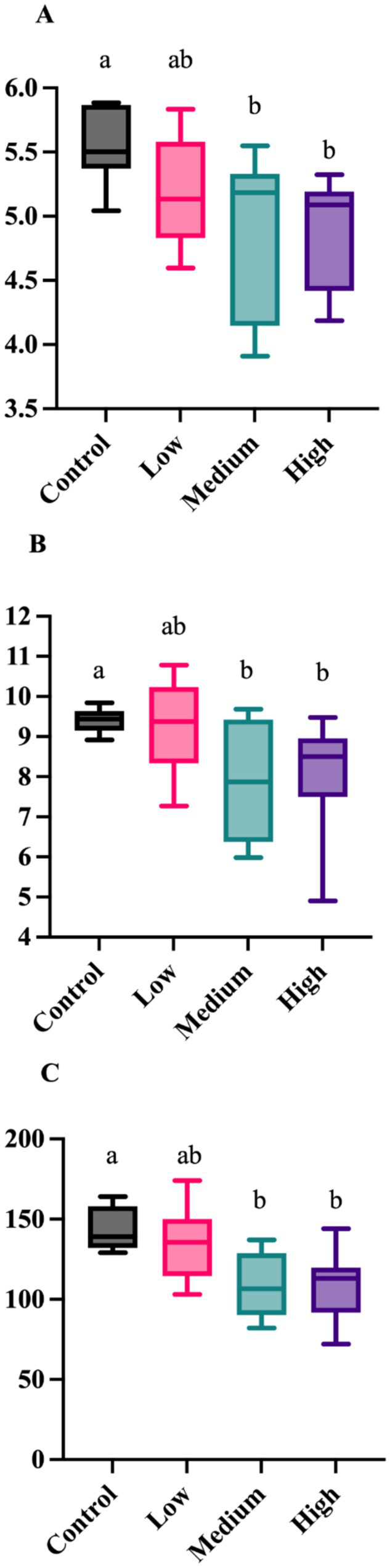
Fecal alpha diversity measures of healthy adult dogs after consuming test diets for 4 wk. The Shannon Diversity Index (**A**; *p* = 0.036), Faith’s phylogenetic diversity (**B**; *p* = 0.031), and observed features (**C**; *p* = 0.009) were greater in dogs fed the Control diet than those fed the medium or high diets.

**Figure 2:**
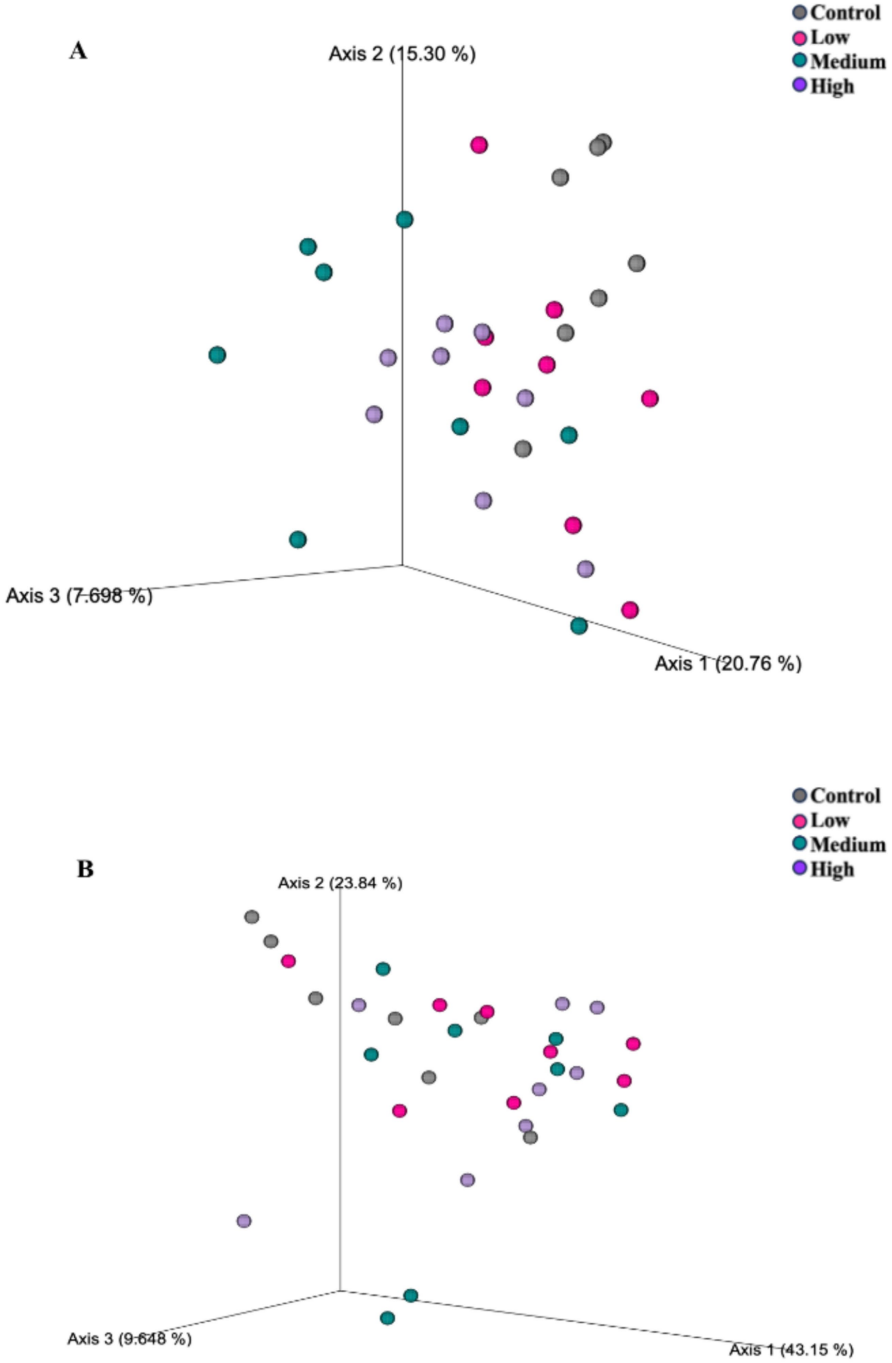
Fecal microbial communities of healthy adult dogs after consuming test diets for 4 wk, as represented as principal coordinates analysis plots of unweighted **(A)** and weighted **(B)** UniFrac distances measures. Each dot represents a sample collected from each dog (*n* = 8/treatment). The unweighted UniFrac distances revealed differences (**A**; *p* = 0.005) among dogs fed the Low diet and Medium diet, and differences (**A**; *p* = 0.005) in dogs fed the Control diet than dogs fed the High and Medium diet. The weighted UniFrac distances revealed differences (**B**; *p* = 0.031) among dogs fed the Control diet than dogs fed the other diets.

After 4 wk on treatment, alpha diversity measures (Shannon Diversity Index, Faith’s phylogenetic diversity, observed features) were altered by dietary treatment (Figure 1). Each measure was greater (*p* < 0.05) in dogs fed the Control diet than those fed the Medium or High diets. The unweighted UniFrac distances revealed differences (*p* = 0.005) among dogs fed the Low diet and Medium diet, and differences (*p* = 0.005) in dogs fed the Control diet than dogs fed the High and Medium diet (Figure 2). The weighted UniFrac distances revealed differences (*p* = 0.031) among dogs fed the Control diet than dogs fed the other diets.

After 4 wk on treatment, 2 fecal bacterial phyla and 21 fecal bacterial genera were altered by dietary treatment group (Table 4). At the phyla level, the relative abundances of Fusobacteriota and Proteobacteria in dogs fed the Control diet were greater (*p* < 0.01) than dogs fed the Medium and High diets. Within the Actinobacteriota phylum, the relative abundance of *Adlercreutzia* was lower (*p* = 0.0066) in dogs fed the Control diet than in dogs fed the other diets. Within the same phylum, the relative abundance of *Slackia* tended to be different (*p* = 0.0860) among dietary treatments groups. Within the Bacteroidota phylum, the relative abundance of *Parabacteroides* was greater (*p* = 0.0043) in dogs fed the High diet than dogs fed the Control and Medium diets. The relative abundance of *Prevotella* was greater (*p* = 0.0476) in dogs fed the Medium and High diets than dogs fed the Low diet. The relative abundance of Prevotellaceae was greater (*p* = 0.0186) in dogs fed the Control diet than dogs fed the Low and Medium diets. In addition, the relative abundance of Muribaculaceae tended to be different (*p* = 0.0753) among dietary treatment groups.

**Table 4:** Bacterial phyla and genera (% of total sequences) in feces of healthy adult dogs after consuming test diets for 4 wk.

Phyla	Genus	Control	Low	Medium	High	SEM	*P-*value
Actinobacteriota		0.62	1.30	3.06	2.84	1.05	0.6225
	*Adlercreutzia*	0.02^b^	0.05^a^	0.05^a^	0.06^a^	0.01	0.0066
	*Bifidobacterium*	0.66	0.90	2.67	2.44	1.02	0.8374
	*Collinsella*	0.06	0.04	0.11	0.14	0.07	0.7904
	Coriobacteriaceae	0.22	0.55	1.22	0.61	0.34	0.3272
	*Slackia*	0.04	0.06	0.03	0.02	0.01	0.0860
Bacteroidota		17.69	15.44	12.68	14.89	2.32	0.5369
	*Alloprevotella*	1.72	2.13	1.02	1.55	0.38	0.2207
	*Bacteroides*	13.10	8.84	8.18	9.27	1.78	0.2639
	Muribaculaceae	0.25	2.92	0.89	0.53	0.64	0.0753
	*Parabacteroides*	0.31^b^	1.13^ab^	0.46^b^	1.85^a^	0.27	0.0043
	*Prevotella*	0.82^ab^	0.58^b^	2.34^a^	2.26^a^	0.48	0.0476
	Prevotellaceae	1.49^a^	0.41^b^	0.33^b^	0.35^ab^	0.26	0.0186
	Rikenellaceae	0.12	0.13	0.07	0.14	0.03	0.5403
Firmicutes		62.28	68.42	76.02	72.41	3.79	0.1069
	*Allobaculum*	1.76	5.98	5.56	2.48	2.16	0.7987
	*Blautia*	2.49	2.83	3.86	3.91	0.77	0.3570
	*Butyricioccus*	0.06	0.07	0.08	0.09	0.01	0.2435
	*Catenisphaera*	0.51	0.15	0.44	0.01	0.42	0.4052
	*Cellulosilyticum*	0.15	0.21	0.32	0.10	0.12	0.6432
	*Clostridium*	1.62^w^	1.30^w^	0.31^x^	2.08^wx^	0.89	0.0002
	*Dubosiella*	1.14	6.36	2.89	3.22	13.94	0.3267
	*Epulopiscium*	0.17	0.18	0.03	0.39	0.12	0.6762
	*Enterococcus*	0.30	0.27	0.13	0.17	0.06	0.2455
	*Erysipelatoclostridium*	0.83^c^	2.56^b^	3.31^ab^	5.36^a^	0.66	<0.0001
	Erysipelotrichaceae uncultured	4.17	4.68	6.71	4.91	1.88	0.7546
	Eubacteriaceae unclassified	0.13	0.07	0.18	0.08	0.03	0.1339
	*Eubacterium*	0.60^a^	0.59^a^	0.24^b^	0.29^ab^	0.09	0.0195
	*Faecalibacterium*	4.76^a^	1.51^b^	2.24^b^	0.33^b^	0.62	0.0004
	*Faecalibaculum*	2.38	9.66	4.59	12.05	3.17	0.2121
	*Faecalitalea*	0.09	0.48	0.21	0.32	0.26	0.6404
	*Fournierella*	0.22	0.16	0.08	0.64	0.29	0.8123
	*Holdemanella*	0.19	0.97	4.78	1.93	1.28	0.2781
	*Intestinimonas*	0.02	0.02	0.02	0.02	0.01	0.5647
	*Lachnoclostridium*	0.54	0.26	0.12	0.17	0.12	0.0611
	Lachnospiraceae	0.23	0.19	0.14	0.20	0.07	0.3443
	Lachnospiraceae unclassified	2.05^a^	0.64^b^	1.09^ab^	0.57^b^	0.24	0.0011
	Lachnospiraceae uncultured	0.84	0.86	0.65	0.80	0.25	0.8816
	*Lactobacillus*	16.32	13.56	14.85	13.35	5.30	0.7932
	*Megamonas*	0.38	0.30	2.53	1.07	1.00	0.1110
	*Negativibacillus*	0.18	0.12	0.07	0.05	0.03	0.1522
	*Oribacterium*	0.05	0.05	0.04	0.05	0.01	0.9655
	Oscillospiraceae	0.09	0.11	0.07	0.07	0.02	0.3035
	*Peptoclostridium*	7.21	5.62	4.32	3.54	1.31	0.1869
	*Peptococcus*	0.29	0.26	0.16	0.19	0.05	0.2547
	*Peptostreptococcus*	1.01	1.09	0.56	0.26	0.42	0.4521
	*Phascolarctobacterium*	1.01	0.76	0.84	0.94	0.15	0.5127
	*Romboutsia*	3.31^a^	2.35^ab^	0.97^b^	1.28^b^	0.49	0.0065
	Ruminococcaceae	0.01	0.01	0.06	0.04	0.03	0.5322
	*Ruminococcus gauvreauii*	0.22^b^	0.42^b^	0.78^ab^	1.37^a^	0.16	0.0002
	*Ruminococcus gnavus*	0.61	0.34	0.55	0.70	0.20	0.5890
	*Ruminococcus torques*	1.90^a^	0.73^b^	0.81^b^	0.35^b^	0.21	0.0002
	*Ruminococcus uncultured*	0.10	0.08	0.05	0.05	0.02	0.0669
	*Sellimonas*	0.48^a^	0.27^ab^	0.20^b^	0.14^b^	0.04	<0.0001
	*Stoquefichus*	0.33	0.60	0.02	0.31	0.23	0.0975
	*Streptococcus*	1.32	1.04	12.81	8.79	3.82	0.1310
	*Terrisporobacter*	0.62	0.36	0.15	1.19	0.32	0.0951
	*Turicibacter*	2.22	2.17	1.82	1.93	0.70	0.8899
Fusobacteriota		14.33^a^	11.38^ab^	6.07^b^	7.34^b^	1.69	0.0092
	*Fusobacterium*	14.33^a^	11.38^ab^	6.07^b^	7.34^b^	1.69	0.0092
Proteobacteria		5.03^a^	3.44^ab^	2.16^b^	2.48^b^	0.51	0.0036
	*Anaerobiospirillum*	1.03	0.50	0.15	0.30	0.23	0.0946
	*Parasutterella*	1.64	2.07	1.08	1.32	0.39	0.3398
	*Sutterella*	2.28	0.92	1.07	0.65	0.39	0.1092

^a,b^Means within a row lacking a common superscript differ (*P* < 0.05) using mixed models procedure.

^w,x,y,z^Means within a row lacking a common superscript differ (*P* < 0.05) using npar1way procedure.

Within the Firmicutes phylum, the relative abundance of *Clostridium* was greater (*p* = 0.0002) in dogs fed the Control and Low diets than dogs fed the Medium diet. The relative abundance of *Erysipelatoclostridium* was greater (*p* < 0.0001) in dogs fed the High diet than dogs fed the Low and Control diet, and greater (*p* < 0.0001) in dogs fed the Medium and Low diets than dogs fed the Control diet. The relative abundance of *Eubacterium* was greater (*p* = 0.0195) in dogs fed the Control and Low diet than dogs fed the Medium diet. The relative abundance of *Faecalibacterium* was greater (*p* = 0.0004) in dogs fed the Control than dogs fed the other diets. The relative abundance of Lachnospiraceae unclassified was greater (*p* = 0.0011) in dogs fed the Control diet than dogs fed the High and Low diets. The relative abundance of *Romboutsia* was greater (*p* = 0.0065) in dogs fed the Control diet than dogs fed the Medium and High diets. The relative abundance of *Ruminococcus gauvreauii* was greater (*p* = 0.0002) in dogs fed the High diet than dogs fed the Control and Low diets. The relative abundance of *Ruminococcus torques* was greater (*p* = 0.0002) in dogs fed the Control diet than dogs fed the other diets. The relative abundance of *Sellimonas* was greater (*p* < 0.0001) in dogs fed the Control diet than dogs fed the Medium and High diets. The relative abundances of *Lachnoclostridium* (*p* = 0.0611), *Ruminococcus uncultured* (*p* = 0.0669), *Stoquefichus* (*p* = 0.0975), and *Terrisporobacter* (*p* = 0.0951) tended to be different among dietary treatment groups. Within the Fusobacteriota phylum, the relative abundance of *Fusobacterium* was greater (*p* = 0.0092) in dogs fed the Control diet than dogs fed the Medium and High diets. Lastly, within the Proteobacteria phylum, the relative abundance of *Anaerobiospirillum* tended to be different (*p* = 0.0946) among treatment groups. Significant linear contrasts were identified for 2 phyla and 11 genera. While fecal *Parabacteroides*, *Prevotella*, *Erysipelatoclostridium*, and *Ruminococcus gauvreauii* were linearly increased (*p* < 0.05), Prevotellaceae, *Eubacterium*, *Faecalibacterium*, *Romboutsia*, *Ruminococcus torques*, *Sellimonas*, Fusobacteriota, *Fusobacterium*, and Proteobacteria were linearly decreased (*p* < 0.001) with increasing dietary BCP inclusion.

After 4 wk on treatment, none of the serum chemistry measures were different among treatment groups (*p* > 0.10; Supplementary Table 5). Most serum chemistry measures were within the reference ranges for adult dogs. However, serum globulin concentrations were below and albumin: globulin ratios were above the reference ranges for all treatment groups. In addition, the Na: K ratios were above the reference range for dogs fed the Control and Medium diets. Serum cytokine and IgE concentrations were different among groups after 4 wk (Supplementary Table 5). Serum IgE concentrations were greater (*p* = 0.0466) in dogs fed the High diet than dogs fed the Medium diet. Serum TNF-alpha concentrations tended to be affected (*p* = 0.0765) by treatment group. Hematology measures and urine characteristics were not different among groups, with all being within reference ranges for adult dogs (Supplementary Table 6).

After 26 wk on treatment, fecal DM % was greater (*p* = 0.0003) in dogs fed the Control and Low diets than those fed the High diet (Table 5). A significant linear contrast was also noted, with fecal DM % linearly decreasing (*p* < 0.0001) with increasing dietary BCP inclusion. Body condition score, BW, food and caloric intake, fecal scores, fecal pH, and fecal IgA concentrations were not different among treatment groups.

**Table 5:** Body condition scores, body weight, food and caloric intake, fecal characteristics, and fecal IgA of healthy adult dogs after consuming test diets for 26 wk.

Item	Control	Low	Medium	High	SEM	*P*-value
Body condition score^1^	5.11	5.21	5.08	5.18	0.12	0.8461
Body weight (BW), kg^1^	9.54	9.44	9.10	9.39	0.38	0.8624
Food intake						
Food, g/d (as-is)^1^	202.12	181.50	189.25	176.75	16.30	0.6962
Dry matter (DM), g/d	185.50	167.50	174.63	161.37	14.99	0.6707
Food intake (DM, g/d)/BW (kg)	37.00	32.50	34.38	31.75	3.61	0.7085
Caloric intake, kcal/d^2^	598.87	565.87	588.50	542.62	0.08	0.8003
Caloric intake (kcal)/BW (kg)^2^	64.00	61.38	64.75	59.25	6.57	0.8188
Fecal characteristics						
Fecal score^3^	2.81	2.69	2.88	2.94	0.13	0.4476
Fecal pH	6.60	6.65	6.56	6.23	0.28	0.7150
Fecal DM, %	32.67^a^	33.39^a^	29.89^ab^	27.02^b^	0.99	0.0003
Fecal IgA, mg/g	4.88	4.83	5.54	5.83	0.50	0.4159

^1^Averages from wk 5 to wk 26.

^2^Metabolizable energy estimated using modified Atwater factors = (3.5 × crude protein) + (8.5 × crude fat) + (3.5 x nitrogen-free extract).

^3^Fecal scores: 1 = hard, dry pellets, small hard mass; 2 = hard, formed, dry stool, remains firm and soft; 3 = soft, formed, and moist stool; retains shape; 4 = soft, unformed stool, assumed shape of containers; 5 = watery, liquid that can be poured.

^a-d^Means within a row with different superscripts differ (*p* < 0.05).

**Figure 3:**
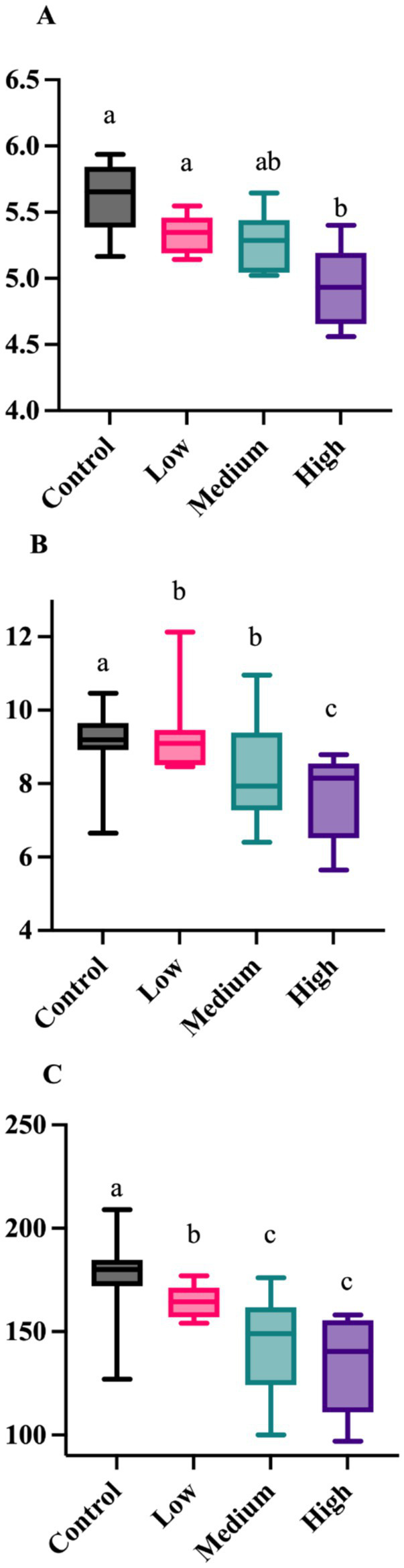
Fecal alpha diversity measures of healthy adult dogs after consuming test diets for 26 wk. The Shannon Diversity Index was greater (**A**; *p* = 0.001) in dogs fed the Control and Low diets than those fed the High diet. Faith’s phylogenetic diversity was greater (**B**; *p* = 0.021) in dogs fed the Control diet than those fed all other diets, and greater (**B**; *p* = 0.021) in dogs fed the Low diet than those fed the Medium and High diets. Observed features were greater (**C**; *p* = 0.002) in dogs fed the Control diet than dogs fed all other diets, and greater (**C**; *p* = 0.002) in dogs fed the Low and Medium diets than those fed the High diet.

**Figure 4:**
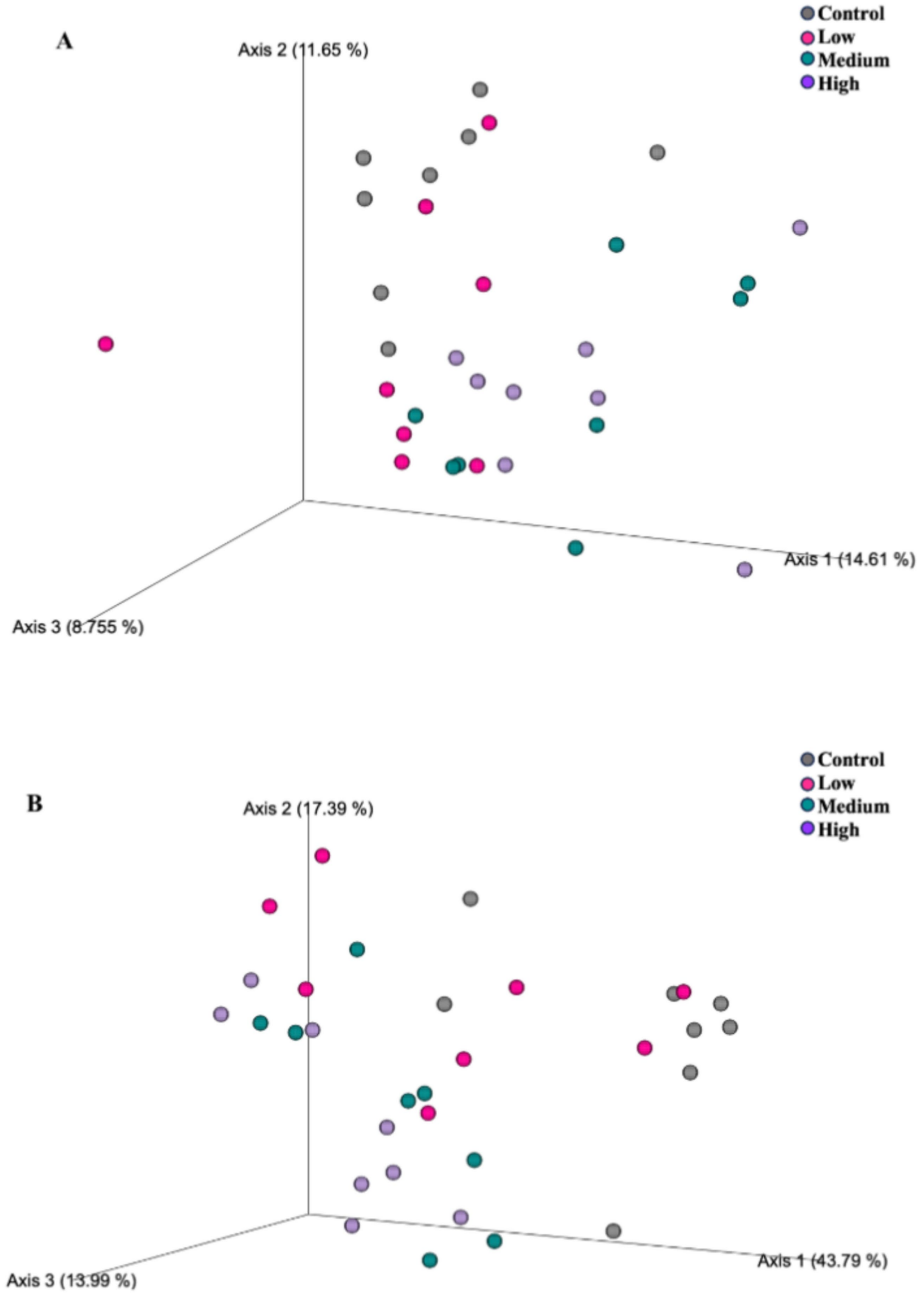
Fecal microbial communities of healthy adult dogs after consuming test diets for 26 wk, as represented by principal coordinates analysis plots of unweighted **(A)** and weighted **(B)** UniFrac distances. Each dot represents a sample collected from each dog (*n* = 8/treatment). The unweighted UniFrac distances revealed that dogs fed the Low diet were different (**A**; *p* = 0.001) than dogs fed the other diets. The weighted UniFrac distances revealed differences (**B**; *p* = 0.001) of dogs fed the control diet from dogs fed the medium and high diets.

After 26 wk on treatment, bacterial alpha diversity indices were again affected by dietary treatment group (Figure 3). The Shannon Diversity Index was greater (*p* = 0.001) in dogs fed the Control and Low diets than those fed the High diet. Faith’s phylogenetic diversity was greater (*p* = 0.021) in dogs fed the Control diet than those fed all other diets, and greater (*p* = 0.021) in dogs fed the Low and Medium diets than those fed the High diet. Observed features were greater (*p* = 0.002) in dogs fed the Control diet than dogs fed all other diets, and greater (*p* = 0.002) in dogs fed the Low diet than those fed the Medium and High diets. Fecal bacterial beta diversity was also affected by dietary treatment (Figure 4). The unweighted UniFrac distances revealed that dogs fed the Low diet were different (*p* = 0.001) than dogs fed the other diets. The weighted UniFrac distances revealed that dogs fed the Control diet were different (*p* = 0.001) than dogs fed the Medium and High diets.

After 26 wk on treatment, the relative abundances of 4 bacterial phyla and nearly 30 bacterial genera were impacted by dietary treatment (Table 6). At the phyla level, the relative abundances of Actinobacteriota (*p* = 0.0046) and Firmicutes (*p* = 0.0115) were greater in dogs fed the Medium and High diets than dogs fed the Control diet. The relative abundance of Fusobacteriota was greater (*p* < 0.0001) in dogs fed the Low and Control diets than in dogs fed the Medium and High diets. The relative abundance of Proteobacteria was greater (*p* = 0.0327) in dogs fed the Control diet than in dogs fed the High and Medium diets.

**Table 6:** Predominant bacterial phyla and genera (% of total sequences) in feces of healthy adult dogs after consuming test diets for 26 wk.

Phyla	Genus	Control	Low	Medium	High	SEM	*P-*value
Actinobacteriota		0.25^b^	0.59^ab^	1.50^a^	1.29^a^	0.25	0.0046
	*Adlercreutzia*	0.02	0.06	0.03	0.01	0.02	0.5054
	*Bifidobacterium*	0.87	0.79	2.44	1.73	0.65	0.5558
	*Collinsella*	0.58	0.55	1.66	1.53	0.36	0.1319
	Coriobacteriaceae	0.13	0.83	1.08	0.64	0.34	0.1731
	*Slackia*	0.10	0.05	0.07	0.05	0.02	0.3200
Bacteroidota		2.71	2.58	2.44	2.45	0.13	0.4313
	*Alloprevotella*	2.10^a^	1.46^ab^	0.98^ab^	0.60^b^	0.36	0.0090
	*Bacteroides*	11.27	9.69	8.39	8.91	1.54	0.4617
	Muribaculaceae	0.27	1.50	0.40	0.34	0.43	0.2958
	*Parabacteroides*	0.35	1.10	0.30	0.96	0.26	0.0583
	*Prevotella*	0.43^b^	0.48^ab^	1.52^a^	1.24^ab^	0.28	0.0212
	Prevotellaceae	0.90	0.36	0.29	0.15	0.22	0.3361
	Rikenellaceae	0.15	0.07	0.02	0.05	0.03	0.0690
Firmicutes		63.42^b^	66.38^ab^	74.68^a^	75.77^a^	2.90	0.0115
	*Allobaculum*	2.28	4.39	4.39	2.53	1.20	0.6918
	*Blautia*	3.25	2.50	5.12	3.66	0.74	0.1106
	*Butyricioccus*	0.07	0.10	0.11	0.11	0.02	0.4849
	*Catenisphaera*	0.21	0.14	0.60	0.27	0.30	0.9063
	*Cellulosilyticum*	0.22	0.23	0.06	0.03	0.10	0.6691
	*Clostridium*	1.85^a^	1.64^ab^	0.48^b^	0.54^ab^	0.38	0.0147
	*Dubosiella*	1.27	4.12	2.29	2.77	1.38	0.6284
	*Enterococcus*	0.33	0.23	0.05	0.30	0.15	0.7140
	*Erysipelatoclostridium*	0.65^b^	3.95^a^	3.77^a^	5.71^a^	1.03	<0.0001
	Erysipelotrichaceae uncultured	3.84	6.59	5.38	4.90	1.76	0.7425
	Eubacteriaceae unclassified	0.04	0.04	0.05	0.03	0.02	0.6832
	*Eubacterium*	0.73^w^	0.66^w^	0.46^w^	0.14^x^	0.12	0.0017
	*Faecalibacterium*	5.73^a^	1.12^b^	2.65^b^	0.36^b^	0.69	<0.0001
	*Faecalibaculum*	1.62	7.05	5.38	8.52	2.25	0.1791
	*Faecalitalea*	0.20	0.04	0.00	0.02	0.09	0.4844
	*Fournierella*	0.20^a^	0.10^ab^	0.08^ab^	0.02^b^	0.04	0.0312
	*Holdemanella*	0.15^b^	0.96^ab^	4.41^a^	6.61^a^	1.71	0.0085
	*Intestinimonas*	0.02^a^	0.02^ab^	0.01^ab^	0.00^b^	0.01	0.0099
	*Lachnoclostridium*	0.61^a^	0.21^b^	0.24^ab^	0.05^b^	0.14	0.0179
	Lachnospiraceae	0.01^x^	0.02^wx^	0.05^w^	0.05^wx^	0.01	0.0339
	Lachnospiraceae unclassified	1.96^a^	0.61^b^	1.15^ab^	0.41^b^	0.31	0.0003
	Lachnospiraceae uncultured	0.77	0.55	0.72	0.33	0.14	0.0524
	*Lactobacillus*	11.62	13.16	15.62	15.32	0.70	0.8562
	*Megamonas*	0.32	0.31	1.30	2.09	0.50	0.0528
	*Negativibacillus*	0.32^a^	0.14^ab^	0.03^bc^	0.02^c^	0.06	0.0001
	*Oribacterium*	0.04^ab^	0.06^a^	0.01^c^	0.02^bc^	0.01	0.0002
	Oscillospiraceae	0.20^a^	0.12^ab^	0.06^b^	0.05^b^	0.04	0.0119
	*Peptoclostridium*	7.59	6.30	6.33	4.57	1.22	0.3229
	*Peptococcus*	0.45^a^	0.27^ab^	0.33^a^	0.12^b^	0.09	0.0054
	*Peptostreptococcus*	0.25	0.70	0.18	0.15	0.19	0.2926
	*Phascolarctobacterium*	0.86	0.70	0.79	0.68	0.11	0.6412
	*Romboutsia*	2.78^a^	2.36^ab^	0.92^b^	1.47^ab^	0.45	0.0268
	Ruminococcaceae	0.16	0.05	0.07	0.06	0.03	0.1304
	*Ruminococcus gauvreauii*	0.16^b^	0.50^ab^	0.99^a^	0.91^a^	0.13	0.0004
	*Ruminococcus gnavus*	0.90	0.48	0.50	0.35	0.24	0.4145
	*Ruminococcus torques*	1.81^a^	0.67^b^	1.22^a^	0.34^b^	0.20	<0.0001
	*Ruminococcus uncultured*	0.09^w^	0.10^w^	0.05^wx^	0.03^x^	0.01	0.0004
	*Sellimonas*	0.38^a^	0.24^ab^	0.19^bc^	0.08^c^	0.04	<0.0001
	*Stoquefichus*	0.21	0.42	0.02	0.01	0.14	0.1334
	*Streptococcus*	3.78	0.77	3.33	7.86	0.93	0.2672
	*Terrisporobacter*	0.45	0.41	0.07	0.11	0.13	0.0976
	*Turicibacter*	3.63	2.46	2.75	2.22	1.05	0.9608
Fusobacteriota		14.85^a^	13.05^a^	5.84^b^	6.03^b^	1.36	<0.0001
	*Fusobacterium*	14.85^a^	13.05^a^	5.84^b^	6.03^b^	1.36	<0.0001
Proteobacteria		4.45^a^	3.54^ab^	2.25^b^	1.93^b^	0.47	0.0023
	*Anaerobiospirillum*	1.23	0.30	0.19	0.30	0.27	0.0883
	*Parasutterella*	1.29	2.32	1.23	1.01	0.40	0.2282
	*Sutterella*	1.78^a^	0.88^ab^	0.72^ab^	0.59^b^	0.26	0.0327

^a,b^Means within a row lacking a common superscript differ (*P* < 0.05) using mixed models procedure.

^w,x,y,z^Means within a row lacking a common superscript differ (*P* < 0.05) using npar1way procedure.

Within the Bacteroidota phylum, the relative abundance of *Alloprevotella* was greater (*p* = 0.0090) in dogs fed the Control diet than those fed the High diet. The relative abundance of *Prevotella* was greater (*p* = 0.0212) in dogs fed the Medium diet than dogs fed the Control diet. In addition, the relative abundances of *Parabacteroides* (*p* = 0.0583) and Rikenellaceae (*p* = 0.0690) tended to be different among dietary treatment groups.

Within the Firmicutes phylum, the relative abundance of C*lostridium* was greater (*p* = 0.0147) in dogs fed the Control diet than dogs fed the Medium diet. The relative abundance of *Erysipelatoclostridium* was lower (*p* < 0.0001) in dogs fed the Control diet than in those fed the other diets. The relative abundance of *Eubacterium* was lower (*p* = 0.0017) in dogs fed the High diet than dogs fed the other diets. The relative abundance of *Faecalibacterium* was greater (*p* < 0.0001) in dogs fed the Control diet than dogs fed the other diets. The relative abundance of *Fournierella* was greater (*p* = 0.0313) in dogs fed the Control diet than dogs fed the High diet. The relative abundance of *Holdemanella* was greater (*p* = 0.0085) in dogs fed the Medium and High diets than dogs fed the Control diet. The relative abundance of *Intestinimonas* was greater (*p* = 0.0099) in dogs fed the Control diet than dogs fed the High diet. The relative abundance of *Lachnoclostridium* was greater (*p* = 0.0179) in dogs fed the Control diet than dogs fed the Low and High diets. The relative abundance of Lachnospiraceae was greater (*p* = 0.0339) in dogs fed the Medium diet than those fed the Control diet. The relative abundance of Lachnospiraceae unclassified was greater (*p* = 0.0003) in dogs fed the Control diet than dogs fed the Low and High diets. The relative abundance of *Negativibacillus* was greater (*p* < 0.0001) in dogs fed the Control diet than dogs fed the Medium and High diets, and greater (*p* < 0.0001) in dogs fed the Low diet than dogs fed the High diet. The relative abundance of *Oribacterium* was greater (*p* = 0.0002) in dogs fed the Low diet than dogs fed the Medium and High diets, and greater (*p* = 0.0002) in dogs fed the Control diet than dogs fed the Medium diet. The relative abundance of Oscillospiraceae was greater (*p* = 0.0119) in dogs fed Control diet than dogs fed the Medium and High diets. The relative abundance of *Peptococcus* was greater (*p* = 0.0054) in dogs fed the Control and Medium diets than dogs fed the High diet. The relative abundance of *Romboutsia* was greater (*p* = 0.0268) in dogs fed the Control than in dogs fed the Medium diet. The relative abundance of *Ruminococcus gauvreauii* was greater (*p* = 0.0004) in dogs fed the Medium and High diets than dogs fed the Control diet. The relative abundance of *Ruminococcus torques* was greater (*p* < 0.0001) in dogs fed the Control and Medium diets than dogs fed the Low and High diets. The relative abundance of *Ruminococcus uncultured* was greater (*p* = 0.0004) in dogs fed the Control and Low diets than dogs fed the High diet. The relative abundance of *Sellimonas* was greater (*p* < 0.0001) in dogs fed the Control diet than dogs fed the Medium and High diets, and greater (*p* < 0.0001) in dogs fed the Low diet than dogs fed the High diet. In addition, the relative abundances of Lachnospiraceae uncultured (*p* = 0.0524), *Megamonas* (*p* = 0.0528), and *Terrisporobacter* (*p* = 0.0976) tended to be different among dietary treatment groups.

Within the Fusobacteriota phylum, the relative abundance of *Fusobacterium* was greater (*p* < 0.0001) in dogs fed the Control and Low diets than dogs fed the Medium and High diets. Within the Proteobacteria phylum, the relative abundance of *Sutterella* was greater (*p* = 0.0327) in dogs fed the Control diet than dogs fed the High diet. Lastly, the relative abundance of *Anaerobiospirillum* (*p* = 0.0883) tended to be different among dietary treatment groups. Significant linear contrasts were identified for 4 phyla and 23 genera. At the phyla level, Actinobacteriota and Firmicutes were linearly increased (*p* < 0.01), while Fusobacteriota and Proteobacteria were linearly decreased (*p* < 0.001) with increasing dietary BCP inclusion. At the genus level, *Prevotella*, *Erysipelatoclostridium*, *Holdemanella*, Lachnospiraceae, and *Ruminococcus gauvreauii* were linearly increased (*p* < 0.05), while *Alloprevotella*, *Clostridium*, *Eubacterium*, *Faecalibacterium*, *Fournierella*, *Intestinimonas*, *Lachnoclostridium*, Lachnospiraceae unclassified, *Negativibacillus*, *Oribacterium*, Oscillospiraceae, *Peptococcus*, *Romboutsia*, *Ruminococcus torques*, *Ruminococcus* uncultured, *Sellimonas*, *Fusobacterium*, and *Sutterella* were linearly decreased (*p* < 0.001) with increasing dietary BCP inclusion.

After 26 wk on treatment, serum corticosteroid-induced ALP concentrations tended to be affected by treatment group (Supplementary Table 7). Serum globulin concentrations and the serum albumin: globulin ratios were again above the reference ranges for adult dogs for all treatment groups. Serum cytokine and IgE concentrations were not different among dietary treatment groups (Supplementary Table 7). Hematology measures and urine characteristics were within the reference ranges for adult dogs and were not different among treatments (Supplementary Table 8).

In the palatability test, a 2.93:1 total consumption ratio was observed for the BCP vs. the control, showing a significant preference (*p* < 0.05) for the diet coated with 1% BCP. Data collected from both days indicate that the 1% BCP was consumed first on 32/40 occasions, compared with 8/40 for the control.

## Discussion

With increasing human and pet populations, a great need for sustainable, high-quality proteins exists. Precision fermentation processes, which incorporate DNA from target animal species into *S. cerevisiae*, have been developed in recent years and may serve as a new source of animal proteins. Before such proteins are approved for use in dog and cat diets, they must undergo adequate testing. To our knowledge, the current study was the first to test a novel brewed chicken protein produced using precision fermentation (e.g., BCP, *S. cerevisiae* expressing a chicken protein) in healthy adult dogs.

Similar to the findings of French et al. (19), BCP inclusion of up to 40% of the diet did not lead to any adverse events. All diets had acceptable palatability and were well-accepted, with all dogs having adequate daily food intake, maintaining BW, and remaining healthy throughout the study. Most of the serum biochemistry analytes remained within the reference ranges for adult dogs. There were a few serum chemistry analytes that were slightly outside the reference range (i.e., globulin; albumin: globulin ratio; Na: K ratio), but these variances were consistent across all treatment groups. Significant differences were observed for serum alkaline phosphatase and alanine transaminase at baseline, but both were within references ranges were not different among groups at wk 4 or 26. Serum IgE concentrations were shown to differ statistically at wk 4, but concentrations were not linearly correlated with increasing dietary BCP (greater in dogs consuming Control and High diets) and no signs of allergy were observed. Moreover, serum IgE concentrations were not different at wk 26 so the occurrence of any allergic response was unlikely. A prior study testing a brewed lamb protein reported no differences in serum IgE concentration at all time points (19). Collectively, these studies suggest that the brewed animal proteins tested did not elicit an allergic response and the statistical differences in IgE were likely not physiologically relevant.

Hematology parameters were within reference ranges for dogs consuming all dietary treatment groups throughout the study. Statistical differences were observed for reticulocyte count, hematocrit, and monocyte % at baseline, but were within the reference ranges, and were not different among diets at wk 4 and 26. Urinalysis, including physical parameters, chemistry, and microscopic sedimentation evaluation were within healthy ranges for all dogs during the study and had no significant differences among dietary treatment groups, also similar to the findings of French et al. (19). Collectively, the lack of change to food intake, BW, serum chemistry, and hematology over 6 mo of feeding suggests that the inclusion of BCP of up to 40% of the diet for adult dogs is safe.

In addition to confirming safety, it is important to test how a novel ingredient impacts the nutrient and energy digestibility of the diet and stool characteristics and fecal output of dogs. In the current study, all dietary treatments were considered to be well-digested by dogs, with the ATTD of most macronutrients being higher than 80%. Despite the lack of differences in food, calorie, and nutrient intake among treatment groups, diets containing BCP had higher digestibilities of DM, organic matter, and protein and consequently reduced fecal output. Yeast proteins have been shown to have high digestibilities. A study conducted by Reilly et al. (33) reported high indispensable amino acid digestibilities (above 80%) for dried yeast. Chicken by-product meal, however, has been shown to have variable digestibilities. The nutrient digestibilities of animal byproducts may fluctuate based on the animal components included (whole or parts of carcass), presence of substances known to impact digestibility (e.g., cartilage), level of processing, and other factors. A study conducted by Oba et al. (34) reported the indispensable amino acid digestibilities of chicken meal at approximately 75% and above. Collectively, these data suggest that the proteins provided by BCP are not only of high quality (amino acid profile), but are also highly digestible.

The inclusion of BCP had the opposite effect on the ATTD of fat, reducing its digestibilty relative to the Control diet. French et al. (19) also reported a lower ATTD of fat in diets containing brewed lamb protein, with a reduction of approximately 10 percentage units (92.4, 85.9, 84.0, and 81.0% ATTD of fat for diets containing 0, 15, 30, and 40% brewed lamb protein, respectively). The reduction was not as extreme in the current study but had a similar trend. A study conducted by Reilly et al. (35) also reported a similar reduction in the ATTD of fat in a diet containing 30% of a dried yeast product (87.9% ATTD of fat for dried yeast diet vs. 94.7% ATTD of fat for control diet containing poultry by-product meal). The presence of phospholipids, sterols (i.e., ergosterol), and sphingolipids within the yeast cell membranes may contribute to the reduction in fat digestibility with dietary BCP inclusion. Phospholipids have been shown to compete with bile acid binding, slightly reducing fat emulsification and digestion; ergosterol also modifies lipid metabolism and absorption by reducing cholesterol absorption and modulating triglyceride digestion (36). The lower fat digestibility with dietary BCP inclusion may also be due to the *β*-glucans or mannanoligosaccharides present within the yeast cell walls. These soluble fibers have been shown to decrease gastric emptying time, increase viscosity, bind bile acids, and reduce nutrient digestibility (37–39).

Fecal DM was reduced with dietary BCP inclusion, but without negative changes to fecal scores. Even though they are somewhat subjective, fecal scores are a good indicator of fecal quality and are used to evaluate consistency (40, 41). The feces of dogs in the current study were soft, formed, and moist and of acceptable quality (3 on 5-point scale), similar to the findings of French et al. (19). These results also suggest that in addition to providing highly digestible protein, the soluble fibers such as *β*-glucans and mannanoligosaccharides that BCP contains can bind to and hold onto water. These presence of these fibers results in the higher moisture content of feces but maintains stool quality (37–39).

Even though stool quality was maintained, fecal metabolites and microbiota were shifted by dietary BCP inclusion. The production of SCFA is a result of dietary fibers and other non-digestible carbohydrates being degraded by saccharolytic bacteria in the large intestine (42). The *β*-glucans and mannanoligosaccharides within the yeast cell wall are readily fermented in the large intestine and serve as a fuel source for microbial populations. Their fermentation often results in greater fecal SCFA concentrations that contribute to intestinal and host health by serving as energy substrates for colonic epithelial cells, maintaining epithelial barrier, regulating energy metabolism, and providing anti-inflammatory effects (15, 43). Fecal total SCFA concentrations were not affected, but acetate and propionate concentrations shifted with dietary BCP inclusion. Acetate is typically the most abundant SCFA produced (44), which was true in the current study. Dogs fed the Control diet had greater acetate concentrations than those containing BCP, which was opposite to that reported by Reilly et al. (35). Reilly et al. (35) evaluated diets containing either garbanzo beans (43.6% of diet), green lentils (44.7% of diet), peanut flour (28.1% of diet), a dried yeast product (29.9% of diet), or poultry by-product meal (33.5% of diet) as the primary protein source. Results from that study showed that fecal acetate concentrations were greater in dogs fed garbanzo beans (368.1 μmol/g) than dogs fed all treatments, and greater in dogs fed green lentils (368.1 μmol/g), peanut flour (361.6 μmol/g), and dried yeast product (349.7 μmol/g) compared with dogs fed the control diet (221.1 μmol/g). In that study, fecal propionate concentrations were greater in dogs fed the garbanzo beans (207.2 μmol/g) than dogs fed all treatments, with dogs fed green lentils (198.5 μmol/g), peanut flour (172.3 μmol/g), and dried yeast product (150.4 μmol/g) being similar to dogs fed the control diet (114.8 μmol/g). In the current study, a linear trend was observed for fecal propionate concentrations (154.69, 234.48, and 247.59 μmol/g for 15, 30, 40% inclusions, respectively), with dogs fed high BCP inclusion being much higher than controls (169.98 μmol/g). Butyrate is recognized for its role in reducing inflammation, regulating the epithelial barrier function, and providing the main energy source for intestinal epithelial cells (7). Reilly et al. (35) reported that fecal butyrate concentrations were greater in dogs fed dried yeast product (150.4 μmol/g) than those fed garbanzo beans (44.4 μmol/g), green lentils (49.7 μmol/g), peanut flour (45.4 μmol/g), and controls (45.2 μmol/g). Dogs in the current study had the same pattern (135.1, 159.2, 183.3 μmol/g for 15, 30, 40% inclusions, respectively, compared with 118.14 μmol/g for dogs fed the Control), but the results were not significantly different. Lastly, Reilly et al. (35) reported total fecal SCFA were lower in dogs fed the control diet (381.1 μmol/g) than dogs fed garbanzo beans (771.0 μmol/g), green lentils (616.2 μmol/g), peanut flour (579.3 μmol/g), and dried yeast product (604.0 μmol/g); however, differences in fecal total SCFA concentrations were not observed in the current study.

Proteolytic fermentation also takes place in the large intestine, resulting in the production of BCFA from the deamination of branched-chained amino acids (i.e., leucine, isoleucine, and valine), the production of phenols and indoles from deaminated aromatic amino acids (i.e., histidine, phenylalanine, tryptophan, and tyrosine), and the production of ammonia from urea, nitrate reduction and amino acid deamination. These catabolites are associated with fecal odor, may be toxic to the intestinal mucosa at high concentrations, and increase luminal pH that favors the survival of pathogenic bacteria, yet the threshold between functional and toxic concentrations of these compounds for dogs remains unknown (7). In the current study, dogs fed the Control diet had higher fecal isobutyrate and isovalerate concentrations than dogs fed the Medium and High diets, and dogs fed the Low diet had higher concentrations than the dogs fed the Medium diet. Additionally, fecal indole, total phenol and indole, and ammonia concentrations were greater in dogs fed the Control and Low diets than those fed the High and Medium diets. The concentrations of fecal valerate, a SCFA derived from amino acid fermentation, had the opposite pattern. Fecal valerate concentrations were higher in dogs fed the High and Medium diets than those fed the Low and Control diets, which is in line with that of Reilly et al. (35) who reported greater valerate concentrations in dogs fed dried yeast product (3.6 μmol/g) than those fed the control diet (1.0 μmol/g). The findings of the current study suggest that while the digestible amino acid content of the chicken by-product meal and BCP were unknown, the BCP may have beneficial effects on the functionality of microbes present in the canine colon.

In addition to the alterations in fecal fermentative metabolite concentrations, the gastrointestinal microbiota populations and diversity indices were modulated by dietary BCP inclusion. These microbial shifts may not only have an impact on gastrointestinal physiology, but also on host metabolism and immunity (45). Dogs consuming dietary BCP had reduced richness and evenness of microbiota, with the relative abundances of Fusobacteriota and Proteobacteria being lower and Actinobacteriota and Firmicutes being higher in dogs fed BCP. These responses are typical of greater fiber fermentation, which may reduce opportunistic pathogens and risk of gastrointestinal dysbiosis. These findings suggest a shift toward fiber fermentation and a shift away from protein fermentation, aligning with the fecal metabolite data.

Several members of the Firmicutes phylum were altered. The relative abundance of *Prevotella*, a SCFA producer and regulator of the intestinal barrier and immune system, was generally greater in dogs fed BCP, suggesting benefits to the host (46). The relative abundance of *Erysipelatoclostridium* was consistently greater in dogs fed BCP. That bacterial taxon is known to break down protein and carbohydrates, producing acetate and lactate (47). The relative abundance of *Clostridium* was lower in dogs consuming BCP. While some *Clostridium* members break down saccharides and produce SCFA, many are proteolytic, positively correlated with protein intake, and lead to the production of protein catabolites. Again, these findings were similar to that of a previous study in our lab testing a *S. cerevisiae* fermentation product (9). Given the responses above, the strong reduction in *Fusobacterium* relative abundance with BCP consumption was not surprising. Because *Fusobacterium* are proteolytic in nature, their reduction goes along with the reduction in protein catabolites reported in this study and previous studies (48).

In conclusion, our findings indicate that the adult dogs fed diets containing a BCP at inclusion levels of up to 40% had no adverse health effects and may have benefitted from the changes to gastrointestinal microbiota and metabolite concentrations. Body weight, body condition scores, food intake, physical parameters, hematology, serum chemistry, serum cytokine and IgE concentrations, urinalyses, and fecal characteristics were largely unchanged among dietary treatment groups and considered acceptable. The digestibility of the diets, fecal output, and stool quality were either not affected or affected in a positive manner. Even though the ATTD of fat was lower in BCP-based diets, the DM, organic matter, crude protein, and energy ATTD of BCP-based diets were greater. The higher ATTD led to reduced fecal output in dogs eating the high concentrations of BCP. Interesting shifts in fecal fermentative metabolites and microbiota were observed in dogs consuming the BCP, likely due to the soluble fiber provided by the yeast component. Collectively, our results suggest that the BCP ingredient tested in this study is safe for use in foods for adult dogs at up to a 40% inclusion level. Future research may be conducted to evaluate the safety of the test protein in dogs and cats of other life stages.

## Data Availability

The original contributions presented in the study are publicly available. Sequence data has been deposited to NCBI (BioSample accessions SAMN49269378-SAMN49269472).
