# Supplementary material for: Safety, efficacy, gastrointestinal tolerance, and digestibility of brewed chicken protein in healthy adult dogs
Source: Front Vet Sci. 2025 Jul 7;12:1593209. doi: 10.3389/fvets.2025.1593209 (PMC12278293; doi:10.3389/fvets.2025.1593209)
Supplement: Supplementary file 1 [file Data_Sheet_1.docx]

| **Supplementary Table 1**. Baseline body condition scores, body weight, food and caloric intakes, fecal characteristics, and fecal IgA concentrations of healthy adult dogs consuming the Control diet^1^ | | | | | | | |
| --- | --- | --- | --- | --- | --- | --- | --- |
| Item | Control (baseline) | Low (baseline) | Medium (baseline) | | High (baseline) | SEM | *P*-value |
| Body condition score^2,3^ | 5.00 | 5.31 | | 4.88 | 5.50 | 0.41 | 0.7006 |
| Body weight (BW), kg^2^ | 9.29 | 9.38 | | 8.93 | 9.50 | 0.39 | 0.7566 |
| Food and caloric intake |  |  | |  |  |  |  |
| Food, g/d (as-is)^2^ | 198.75 | 195.13 | | 187.25 | 190.75 | 0.04 | 0.6332 |
| Dry matter (DM), g/d | 183.50 | 179.12 | | 172.75 | 175.00 | 0.04 | 0.6180 |
| Food intake (g/d DM)/BW (kg) | 19.71 | 19.21 | | 19.56 | 18.88 | 0.99 | 0.9337 |
| Caloric intake, kcal/d^4^ | 588.90 | 578.16 | | 554.83 | 565.20 | 19.49 | 0.6310 |
| Caloric intake (kcal)/BW (kg)^4^ | 63.60 | 62.00 | | 63.13 | 60.91 | 3.18 | 0.9333 |
| Fecal characteristics |  |  | |  |  |  |  |
| Fecal score^5^ | 2.94 | 2.94 | | 3.00 | 3.06 | 0.07 | 0.5636 |
| Fecal pH | 6.78 | 7.20 | | 6.95 | 7.04 | 0.24 | 0.6645 |
| Fecal DM, % | 32.16 | 33.77 | | 33.48 | 32.43 | 0.96 | 0.5840 |
| Fecal IgA, mg/g | 14.21 | 11.82 | | 12.94 | 14.07 | 1.01 | 0.5772 |
| ^1^All dogs consumed the control diet for 2 wk prior to baseline measurements.  ^2^Averages during acclimation phase.  ^3^9-point scale (Laflamme, 1997).  ^4^Metabolizable energy estimated using modified Atwater factors = (3.5 x crude protein) + (8.5 x crude fat) + (3.5 x nitrogen-free extract).  ^5^Fecal scores: 1 = hard, dry pellets, small hard mass; 2 = hard, formed, dry stool, remains firm and soft; 3 = soft, formed, and moist stool; retains shape; 4 = soft, unformed stool, assumed shape of containers; 5 = watery, liquid that can be poured. | | | | | | | |

| **Supplementary Table 2.** Baseline serum chemistry, cytokines, and IgE concentrations of healthy adult dogs consuming the Control diet^1^ | | | | | | | |
| --- | --- | --- | --- | --- | --- | --- | --- |
| Item | Reference Range^2^ | Control (baseline) | Low (baseline) | Medium (baseline) | High (baseline) | SEM | *P*-value |
| Serum Chemistry |  |  |  |  |  |  |  |
| Creatinine, mg/dL | 0.5–1.5 | 0.58 | 0.69 | 0.64 | 0.61 | 0.04 | 0.2578 |
| Blood urea nitrogen, mg/dL | 6–30 | 13.75 | 16.13 | 15.63 | 14.63 | 0.79 | 0.1675 |
| Total protein, g/dL | 5.1–7.0 | 5.94 | 5.84 | 5.84 | 6.28 | 0.13 | 0.0774 |
| Albumin, g/dL | 2.5–3.8 | 3.34 | 3.26 | 3.34 | 3.39 | 0.08 | 0.7680 |
| Globulin, g/dL | 2.7–4.4 | 2.60 | 2.58 | 2.50 | 2.89 | 0.10 | 0.0607 |
| Albumin: globulin ratio | 0.6–1.1 | 1.30 | 1.28 | 1.35 | 1.19 | 0.06 | 0.2766 |
| Ca, mg/dL | 7.6–11.4 | 9.76 | 9.78 | 9.76 | 9.94 | 0.10 | 0.5357 |
| P, mg/dL | 2.7–5.2 | 3.51 | 3.25 | 3.48 | 4.05 | 0.06 | 0.0930 |
| Na, mmol/L | 141–152 | 146.63 | 146.75 | 146.50 | 146.50 | 0.50 | 0.9792 |
| K, mmol/L | 3.9–5.5 | 3.95 | 4.04 | 4.01 | 4.23 | 0.07 | 0.0525 |
| Na: K ratio | 28–36 | 37.13 | 36.50 | 36.50 | 34.88 | 0.61 | 0.0817 |
| Cl, mmol/L | 107–118 | 112.75 | 113.13 | 112.88 | 112.12 | 0.66 | 0.7448 |
| Glucose, mg/dL | 68–126 | 89.38 | 86.88 | 92.00 | 84.63 | 0.05 | 0.6281 |
| Alkaline phosphatase (ALP), U/L | 7–92 | 45.63^ab^ | 41.63^ab^ | 35.25^b^ | 61.63^a^ | 5.41 | 0.0127 |
| Corticosteroid-induced ALP, U/L | 0–40 | 1.25 | 3.00 | 1.38 | 4.88 | 1.44 | 0.2657 |
| Alanine transaminase, U/L | 8–65 | 26.75^b^ | 35.25^a^ | 27.00^b^ | 27.63^ab^ | 2.08 | 0.0204 |
| Gamma glutamyltransferase, U/L | 0–7 | 3.38 | 3.38 | 3.25 | 3.13 | 3.38 | 0.9227 |
| Total bilirubin, mg/dL | 0.1–0.3 | 0.21 | 0.28 | 0.20 | 0.23 | 0.03 | 0.2770 |
| Creatine phosphokinase, U/L | 26–310 | 123.25 | 119.00 | 103.25 | 151.00 | 17.36 | 0.2903 |
| Cholesterol, mg/dL | 129–297 | 156.50 | 163.50 | 142.63 | 181.63 | 12.29 | 0.1810 |
| Triglycerides, mg/dL | 32–154 | 42.25 | 39.50 | 42.00 | 55.38 | 0.09 | 0.0557 |
| Bicarbonate, mmol/L | 16–24 | 21.25 | 21.00 | 21.38 | 20.00 | 0.67 | 0.4732 |
| Anion gap | 8–25 | 16.63 | 16.63 | 16.25 | 18.63 | 0.69 | 0.0865 |
| Serum cytokines and IgE |  |  |  |  |  |  |  |
| IL-6, pg/mL | --- | 31.85 | 81.50 | 52.90 | 39.83 | 20.84 | 0.3926 |
| TNF-α, pg/mL | --- | 219.92 | 83.91 | 78.08 | 168.48 | 76.81 | 0.3326 |
| IgE, ng/mL | --- | 413.68 | 372.07 | 363.10 | 446.06 | 41.25 | 0.4846 |
| ^1^All dogs consumed the control diet for 2 wk prior to baseline measurements.  ^2^University of Illinois Veterinary Medicine Diagnostics Laboratory Reference Ranges.  ^a-d^Means within a row with different superscripts differ (*P*<0.05). | | | | | | | |

| **Supplementary Table 3.** Baseline complete blood count and urine characteristics of healthy adult dogs consuming the Control diet^1^ | | | | | | | |
| --- | --- | --- | --- | --- | --- | --- | --- |
| Item | Reference Range^2^ | Control (baseline) | Low (baseline) | Medium (baseline) | High (baseline) | SEM | *P*-value |
| Red blood cells, 10^6^/µL | 5.50-8.50 | 7.17 | 7.04 | 6.75 | 7.43 | 0.22 | 0.1937 |
| Reticulocyte count, % | --- | 0.49^b^ | 0.60^b^ | 0.57^b^ | 1.10^a^ | 0.10 | 0.0011 |
| Hemoglobin, g/dL | 12.0-18.0 | 16.29 | 13.80 | 15.28 | 17.14 | 1.01 | 0.1366 |
| Hematocrit, % | 35.0-52.0 | 47.45^ab^ | 46.18^ab^ | 44.54^b^ | 49.85^a^ | 1.25 | 0.0373 |
| Mean cell volume, fl | 58.0-76.0 | 66.23 | 65.66 | 66.10 | 66.59 | 0.55 | 0.7016 |
| Mean corpuscular hemoglobin, pg | 20.0-25.0 | 22.73 | 22.46 | 22.68 | 22.88 | 0.24 | 0.6925 |
| Mean corpuscular hemoglobin, g/dL | 33.0-38.6 | 35.41 | 34.21 | 34.30 | 34.38 | 0.63 | 0.5110 |
| Platelets, 10^3^/µL | 200-700 | 283.13 | 312.00 | 335.50 | 329.25 | 25.96 | 0.4963 |
| White blood cell count, 10^3^/µL | 6.00-17.00 | 7.48 | 7.27 | 7.29 | 7.95 | 0.72 | 0.8984 |
| Lymphocytes, % | --- | 22.65 | 23.71 | 26.16 | 22.91 | 2.40 | 0.7240 |
| Monocytes, % | --- | 5.05^ab^ | 3.39^b^ | 5.39^a^ | 3.56^ab^ | 0.51 | 0.0172 |
| Eosinophils, % | --- | 2.60 | 2.70 | 3.20 | 3.05 | 0.64 | 0.8985 |
| Basophils, % | --- | 0.13 | 0.10 | 0.08 | 0.14 | 0.06 | 0.8809 |
| Lymphocytes, 10^3^/µL | 1.00-4.80 | 1.70 | 1.70 | 1.92 | 1.71 | 0.21 | 0.8603 |
| Monocytes, 10^3^/µL | 0.20-1.40 | 0.39 | 0.25 | 0.38 | 0.28 | 0.05 | 0.1068 |
| Eosinophils, 10^3^/µL | 0.10-1.00 | 0.21 | 0.18 | 0.23 | 0.25 | 0.23 | 0.8747 |
| Basophils, 10^3^/µL | 0.00-2.00 | 0.01 | 0.01 | 0.01 | 0.01 | 0.00 | 0.9321 |
| Urine Characteristics |  |  |  |  |  |  |  |
| Specific gravity | --- | 1.04 | 1.05 | 1.03 | 0.92 | 0.06 | 0.4040 |
| pH | --- | 7.50 | 7.38 | 7.19 | 7.56 | 0.47 | 0.9446 |
| Total protein | --- | 0.94 | 1.00 | 0.19 | 0.88 | 0.39 | 0.4400 |
| ^1^All dogs consumed the control diet for 2 wk prior to baseline measurements.  ^2^University of Illinois Veterinary Medicine Diagnostics Laboratory Reference Ranges.  ^a-d^Means within a row with different superscripts differ (*P*<0.05). | | | | | | | |

| **Supplementary Table 4.** Baseline bacterial phyla and genera (% of total sequences) in feces of healthy adult dogs after consuming the Control diet^1^ | | | | | | | |
| --- | --- | --- | --- | --- | --- | --- | --- |
| Phyla | Genus | Control (baseline) | Low (baseline) | Medium (baseline) | High (baseline) | SEM | *P-*value |
| Actinobacteriota |  | 0.77 | 1.54 | 1.75 | 1.01 | 0.70 | 0.7231 |
|  | *Adlercreutzia* | 0.00 | 0.05 | 0.02 | 0.02 | 0.02 | 0.0946 |
|  | *Bifidobacterium* | 0.68 | 1.22 | 1.58 | 0.85 | 0.64 | 0.9955 |
|  | *Collinsella* | 0.03 | 0.05 | 0.03 | 0.04 | 0.01 | 0.6858 |
|  | Coriobacteriaceae | 0.00 | 0.15 | 0.08 | 0.04 | 0.06 | 0.7993 |
|  | *Slackia* | 0.06 | 0.07 | 0.05 | 0.05 | 0.02 | 0.9371 |
| Bacteroidota |  | 15.53 | 14.90 | 13.97 | 14.47 | 0.16 | 0.8183 |
|  | *Alloprevotella* | 2.00 | 0.81 | 1.88 | 2.74 | 0.68 | 0.8123 |
|  | *Bacteroides* | 10.34 | 8.96 | 10.29 | 9.60 | 1.67 | 0.8610 |
|  | Muribaculaceae | 0.14 | 1.14 | 0.18 | 0.44 | 0.27 | 0.4922 |
|  | *Parabacteroides* | 0.23 | 0.32 | 0.19 | 0.16 | 0.06 | 0.2570 |
|  | *Prevotella* | 1.13 | 0.82 | 0.87 | 0.64 | 0.36 | 0.8159 |
|  | Prevotellaceae | 1.60 | 1.26 | 0.50 | 0.83 | 0.48 | 0.3708 |
|  | Rikenellaceae | 0.09 | 0.18 | 0.06 | 0.05 | 0.05 | 0.7850 |
| Firmicutes |  | 64.28 | 61.61 | 66.45 | 62.34 | 3.70 | 0.7935 |
|  | *Allobaculum* | 1.29 | 5.44 | 2.01 | 1.83 | 0.88 | 0.0769 |
|  | *Anaerofilum* | 0.39 | 0.20 | 0.32 | 0.44 | 0.10 | 0.2285 |
|  | *Blautia* | 4.29 | 3.08 | 3.34 | 2.76 | 0.57 | 0.2886 |
|  | *Butyricioccus* | 0.06 | 0.05 | 0.06 | 0.04 | 0.01 | 0.5922 |
|  | *Catenisphaera* | 0.18 | 0.41 | 0.31 | 0.21 | 0.22 | 0.8237 |
|  | *Cellulosilyticum* | 0.07 | 0.18 | 0.12 | 0.16 | 0.07 | 0.6906 |
|  | *Clostridium* | 1.14 | 0.55 | 0.84 | 1.53 | 0.35 | 0.1981 |
|  | *Dubosiella* | 0.05^b^ | 1.61^a^ | 0.41^ab^ | 1.01^ab^ | 0.68 | 0.0362 |
|  | *Epulopiscium* | 0.03 | 0.07 | 0.08 | 0.27 | 0.13 | 0.9476 |
|  | *Enterococcus* | 0.43 | 0.08 | 0.08 | 0.19 | 0.11 | 0.3764 |
|  | *Erysipelatoclostridium* | 0.78 | 0.41 | 0.69 | 0.74 | 0.15 | 0.3304 |
|  | Erysipelotrichaceae uncultured | 1.16 | 4.68 | 4.32 | 5.05 | 1.90 | 0.7306 |
|  | Eubacteriaceae unclassified | 0.01 | 0.11 | 0.02 | 0.07 | 0.03 | 0.0876 |
|  | *Eubacterium* | 0.53 | 0.80 | 0.46 | 0.41 | 0.18 | 0.4264 |
|  | *Faecalibacterium* | 6.33 | 3.55 | 5.99 | 7.32 | 1.23 | 0.1941 |
|  | *Faecalibaculum* | 0.06 | 1.75 | 1.02 | 2.35 | 0.90 | 0.3961 |
|  | *Faecalitalea* | 0.27 | 0.04 | 0.13 | 0.23 | 0.12 | 0.8388 |
|  | *Fournierella* | 0.29 | 0.17 | 0.29 | 0.26 | 0.07 | 0.0675 |
|  | *Holdemanella* | 0.37 | 0.16 | 0.31 | 0.32 | 0.09 | 0.0857 |
|  | *Intestinimonas* | 0.02 | 0.01 | 0.01 | 0.01 | 0.01 | 0.2579 |
|  | *Lachnoclostridium* | 0.64 | 0.54 | 0.57 | 0.94 | 0.19 | 0.4620 |
|  | *Lachnospira* | 0.32 | 0.18 | 0.49 | 0.23 | 0.09 | 0.2175 |
|  | Lachnospiraceae | 0.49 | 0.26 | 0.31 | 0.36 | 0.09 | 0.2850 |
|  | Lachnospiraceae unclassified | 3.07 | 1.85 | 2.89 | 2.48 | 0.58 | 0.4717 |
|  | Lachnospiraceae uncultured | 0.99 | 0.94 | 1.01 | 1.11 | 0.17 | 0.9173 |
|  | *Lactobacillus* | 13.34 | 13.14 | 20.66 | 9.46 | 5.29 | 0.5105 |
|  | *Megamonas* | 0.38 | 0.31 | 0.48 | 0.28 | 0.12 | 0.6364 |
|  | *Negativibacillus* | 0.19 | 0.33 | 0.16 | 0.15 | 0.10 | 0.4347 |
|  | *Oribacterium* | 0.02 | 0.03 | 0.03 | 0.03 | 0.02 | 0.7806 |
|  | Oscillospiraceae | 0.12 | 0.15 | 0.10 | 0.08 | 0.04 | 0.8511 |
|  | *Peptoclostridium* | 9.21 | 8.98 | 8.39 | 8.28 | 1.28 | 0.9449 |
|  | *Peptococcus* | 0.32 | 0.49 | 0.27 | 0.25 | 0.88 | 0.4865 |
|  | *Peptostreptococcus* | 0.53 | 0.99 | 0.29 | 0.55 | 0.42 | 0.8403 |
|  | *Phascolarctobacterium* | 0.83 | 0.89 | 0.84 | 0.89 | 0.20 | 0.9952 |
|  | *Romboutsia* | 2.78 | 2.70 | 2.83 | 3.29 | 0.69 | 0.7943 |
|  | Ruminococcaceae | 0.10 | 0.04 | 0.05 | 0.07 | 0.02 | 0.2981 |
|  | *Ruminococcus gauvreauii* | 0.19 | 0.15 | 0.10 | 0.12 | 0.03 | 0.2472 |
|  | *Ruminococcus gnavus* | 0.47 | 0.26 | 0.37 | 0.57 | 0.12 | 0.3420 |
|  | *Ruminococcus torques* | 2.63^a^ | 1.47^b^ | 1.84^ab^ | 1.82^ab^ | 0.26 | 0.0268 |
|  | *Ruminococcus uncultured* | 0.09 | 0.07 | 0.10 | 0.09 | 0.02 | 0.6425 |
|  | *Sellimonas* | 0.52 | 0.46 | 0.40 | 0.33 | 0.07 | 0.0832 |
|  | *Stoquefichus* | 0.17 | 0.12 | 0.14 | 0.23 | 0.07 | 0.4824 |
|  | *Streptococcus* | 4.18 | 1.85 | 0.87 | 1.03 | 1.28 | 0.3785 |
|  | *Terrisporobacter* | 0.47 | 0.21 | 0.46 | 1.02 | 0.27 | 0.0875 |
|  | *Turicibacter* | 3.71 | 1.43 | 1.50 | 2.84 | 1.01 | 0.0888 |
| Fusobacteriota |  | 14.66 | 17.35 | 13.31 | 17.17 | 1.61 | 0.2340 |
|  | *Fusobacterium* | 14.66 | 17.35 | 13.31 | 17.17 | 1.61 | 0.2340 |
| Proteobacteria |  | 4.69 | 4.57 | 4.46 | 4.98 | 0.89 | 0.8886 |
|  | *Anaerobiospirillum* | 0.93 | 1.21 | 0.82 | 1.02 | 0.35 | 0.6170 |
|  | *Parasutterella* | 1.40 | 2.14 | 1.76 | 2.03 | 0.47 | 0.6819 |
|  | *Sutterella* | 2.34 | 1.19 | 1.86 | 1.90 | 0.43 | 0.3149 |
| ^1^All dogs consumed the control diet for 2 wk prior to baseline measurements.  ^a,b^Means within a row lacking a common superscript differ (*P*<0.05) using mixed models procedure. | | | | | | | |

| **Supplementary Table 5.** Serum chemistry, cytokines, and IgE concentrations of healthy adult dogs after consuming test diets for 4 wk | | | | | | | |
| --- | --- | --- | --- | --- | --- | --- | --- |
| Item | Reference Range^1^ | Control | Low | Medium | High | SEM | *P*-value |
| Serum chemistry |  |  |  |  |  |  |  |
| Creatinine, mg/dL | 0.5–1.5 | 0.64 | 0.66 | 0.61 | 0.63 | 0.04 | 0.8660 |
| Blood urea nitrogen, mg/dL | 6–30 | 12.88 | 14.50 | 13.38 | 12.63 | 0.87 | 0.4498 |
| Total protein, g/dL | 5.1–7.0 | 5.86 | 5.75 | 5.83 | 5.99 | 0.11 | 0.5262 |
| Albumin, g/dL | 2.5–3.8 | 3.36 | 3.24 | 3.38 | 3.35 | 0.07 | 0.4330 |
| Globulin, g/dL | 2.7–4.4 | 2.50 | 2.51 | 2.45 | 2.64 | 0.12 | 0.7138 |
| Albumin: globulin ratio | 0.6–1.1 | 1.36 | 1.30 | 1.39 | 1.29 | 0.07 | 0.7016 |
| Ca, mg/dL | 7.6–11.4 | 9.86 | 9.84 | 9.93 | 9.91 | 0.10 | 0.9238 |
| P, mg/dL | 2.7–5.2 | 3.69 | 3.79 | 3.83 | 4.19 | 0.07 | 0.5775 |
| Na, mmol/L | 141–152 | 146.25 | 145.38 | 145.75 | 145.75 | 0.37 | 0.4296 |
| K, mmol/L | 3.9–5.5 | 3.94 | 4.06 | 4.00 | 4.10 | 0.08 | 0.4500 |
| Na: K ratio | 28–36 | 37.25 | 35.88 | 36.25 | 35.50 | 0.69 | 0.3365 |
| Cl, mmol/L | 107–118 | 112.37 | 111.50 | 110.87 | 110.63 | 0.63 | 0.2268 |
| Glucose, mg/dL | 68–126 | 87.63 | 82.00 | 88.88 | 85.38 | 0.05 | 0.5800 |
| Alkaline phosphatase (ALP), U/L | 7–92 | 39.13 | 39.13 | 26.38 | 40.38 | 4.88 | 0.1634 |
| Corticosteroid-induced ALP, U/L | 0–40 | 0.63 | 2.62 | 0.75 | 3.38 | 1.16 | 0.2668 |
| Alanine transaminase, U/L | 8–65 | 27.13 | 28.25 | 27.38 | 28.75 | 2.35 | 0.9573 |
| Gamma glutamyltransferase, U/L | 0–7 | 2.88 | 2.63 | 3.50 | 3.13 | 0.25 | 0.1000 |
| Total bilirubin, mg/dL | 0.1–0.3 | 0.19 | 0.29 | 0.24 | 0.24 | 0.03 | 0.1184 |
| Creatine phosphokinase, U/L | 26–310 | 101.38 | 107.75 | 104.38 | 110.75 | 9.02 | 0.8931 |
| Cholesterol, mg/dL | 129–297 | 153.62 | 158.75 | 132.13 | 145.25 | 0.08 | 0.4407 |
| Triglycerides, mg/dL | 32–154 | 44.13 | 38.63 | 42.25 | 47.25 | 0.12 | 0.7309 |
| Bicarbonate, mmol/L | 16–24 | 21.75 | 21.25 | 22.50 | 21.88 | 0.67 | 0.6249 |
| Anion gap | 8–25 | 16.13 | 16.63 | 16.38 | 17.50 | 0.53 | 0.3002 |
| Serum cytokines and IgE |  |  |  |  |  |  |  |
| IL-6, pg/mL | --- | 107.92 | 47.02 | 71.72 | 58.50 | 0.73 | 0.8791 |
| TNF-α, pg/mL | --- | 173.60 | 73.13 | 74.24 | 193.65 | 0.26 | 0.0765 |
| IgE, ng/mL | --- | 396.24^wx^ | 217.29^wx^ | 202.60^x^ | 396.62^w^ | 60.63 | 0.0466 |
| ^1^University of Illinois Veterinary Medicine Diagnostics Laboratory Reference Ranges.  ^a-d^Means within a row with different superscripts differ (*P*<0.05).  ^w,x,y,z^Means within a row lacking a common superscript differ (*P*<0.05) using npar1way procedure. | | | | | | | |

| **Supplementary Table 6.** Complete blood count and urine characteristics of healthy adult dogs after consuming test diets for 4 wk | | | | | | | |
| --- | --- | --- | --- | --- | --- | --- | --- |
| Item | Reference Range^1^ | Control | Low | Medium | High | SEM | *P*-value |
| Complete blood count |  |  |  |  |  |  |  |
| Red blood cells, 10^6^/µL | 5.50-8.50 | 7.07 | 7.14 | 6.66 | 7.15 | 0.21 | 0.3471 |
| Reticulocyte count, % | --- | 0.46 | 0.51 | 0.39 | 0.55 | 0.07 | 0.4607 |
| Hemoglobin, g/dL | 12.0-18.0 | 15.89 | 15.93 | 14.94 | 16.04 | 0.50 | 0.4050 |
| Hematocrit, % | 35.0-52.0 | 46.40 | 46.13 | 43.36 | 47.16 | 1.36 | 0.2560 |
| Mean cell volume, fl | 58.0-76.0 | 65.76 | 64.60 | 65.13 | 65.96 | 0.53 | 0.2967 |
| Mean corpuscular hemoglobin, pg | 20.0-25.0 | 22.49 | 22.30 | 22.41 | 22.43 | 0.22 | 0.9503 |
| Mean corpuscular hemoglobin, g/dL | 33.0-38.6 | 34.23 | 34.51 | 34.44 | 34.01 | 0.19 | 0.3146 |
| Platelets, 10^3^/µL | 200-700 | 280.86 | 312.12 | 280.25 | 265.14 | 24.83 | 0.6179 |
| White blood cell count, 10^3^/µL | 6.00-17.00 | 7.03 | 7.49 | 8.21 | 6.57 | 0.92 | 0.6579 |
| Lymphocytes, % | --- | 28.51 | 25.88 | 28.38 | 30.03 | 2.22 | 0.6390 |
| Monocytes, % | --- | 3.66 | 3.73 | 4.03 | 3.79 | 0.25 | 0.7547 |
| Eosinophils, % | --- | 3.16 | 2.83 | 2.94 | 3.14 | 0.51 | 0.9619 |
| Basophils, % | --- | 0.19 | 0.20 | 0.18 | 0.26 | 0.04 | 0.5631 |
| Lymphocytes, 10^3^/µL | 1.00-4.80 | 1.91 | 1.93 | 1.96 | 1.93 | 0.25 | 0.9987 |
| Monocytes, 10^3^/µL | 0.20-1.40 | 0.26 | 0.28 | 0.33 | 0.25 | 0.04 | 0.5312 |
| Eosinophils, 10^3^/µL | 0.10-1.00 | 0.22 | 0.21 | 0.24 | 0.22 | 0.05 | 0.9769 |
| Basophils, 10^3^/µL | 0.00-2.00 | 0.01 | 0.01 | 0.01 | 0.02 | 0.00 | 0.9498 |
| Urine Characteristics |  |  |  |  |  |  |  |
| Specific gravity | --- | 1.04 | 1.05 | 1.05 | 1.04 | 0.01 | 0.6108 |
| pH | --- | 8.25 | 7.94 | 8.69 | 7.75 | 0.34 | 0.2378 |
| Total protein | --- | 0.75 | 1.06 | 1.19 | 0.81 | 0.31 | 0.7323 |
| ^1^University of Illinois Veterinary Medicine Diagnostics Laboratory Reference Ranges. | | | | | | | |

| **Supplementary Table 7.** Serum chemistry, cytokines, and IgE concentrations of healthy adult dogs after consuming test diets for 26 wk | | | | | | | |
| --- | --- | --- | --- | --- | --- | --- | --- |
| Item | Reference Range^1^ | Control | Low | Medium | High | SEM | *P*-value |
| Serum Chemistry |  |  |  |  |  |  |  |
| Creatinine, mg/dL | 0.5–1.5 | 0.63 | 0.69 | 0.60 | 0.58 | 0.04 | 0.3315 |
| Blood urea nitrogen, mg/dL | 6–30 | 14.63 | 16.25 | 14.75 | 13.38 | 1.06 | 0.3181 |
| Total protein, g/dL | 5.1–7.0 | 6.11 | 5.94 | 5.86 | 6.19 | 0.14 | 0.3569 |
| Albumin, g/dL | 2.5–3.8 | 3.44 | 3.29 | 3.30 | 3.33 | 0.10 | 0.6726 |
| Globulin, g/dL | 2.7–4.4 | 2.68 | 2.65 | 2.56 | 2.86 | 0.11 | 0.2575 |
| Albumin: globulin ratio | 0.6–1.1 | 1.29 | 1.25 | 1.29 | 1.19 | 0.06 | 0.5890 |
| Ca, mg/dL | 7.6–11.4 | 9.70 | 9.83 | 9.69 | 9.58 | 0.12 | 0.5256 |
| P, mg/dL | 2.7–5.2 | 3.48 | 3.28 | 3.71 | 3.76 | 0.26 | 0.5288 |
| Na, mmol/L | 141–152 | 145.75 | 145.38 | 145.75 | 144.88 | 0.47 | 0.5184 |
| K, mmol/L | 3.9–5.5 | 4.08 | 4.15 | 4.00 | 4.14 | 2.53 | 0.9529 |
| Na: K ratio | 28–36 | 35.88 | 35.00 | 36.50 | 35.38 | 0.69 | 0.4607 |
| Cl, mmol/L | 107–118 | 112.25 | 111.62 | 111.62 | 111.00 | 0.62 | 0.3020 |
| Glucose, mg/dL | 68–126 | 88.38 | 86.50 | 89.25 | 88.38 | 4.03 | 0.9689 |
| Alkaline phosphatase (ALP), U/L | 7–92 | 33.75 | 33.88 | 23.38 | 41.63 | 23.53 | 0.9930 |
| Corticosteroid-induced ALP, U/L | 0–40 | 1.13 | 2.38 | 0.88 | 3.50 | 1.07 | 0.0827 |
| Alanine transaminase, U/L | 8–65 | 28.00 | 28.63 | 28.50 | 31.13 | 2.94 | 0.8766 |
| Gamma glutamyltransferase, U/L | 0–7 | 2.75 | 2.88 | 2.88 | 3.00 | 0.29 | 0.9743 |
| Total bilirubin, mg/dL | 0.1–0.3 | 0.21 | 0.30 | 0.28 | 0.26 | 0.04 | 0.4062 |
| Creatine phosphokinase, U/L | 26–310 | 112.63 | 105.37 | 112.50 | 123.63 | 14.07 | 0.8919 |
| Cholesterol, mg/dL | 129–297 | 150.75 | 167.50 | 132.75 | 154.12 | 10.12 | 0.1364 |
| Triglycerides, mg/dL | 32–154 | 38.88 | 37.13 | 39.88 | 45.38 | 0.85 | 0.3894 |
| Bicarbonate, mmol/L | 16–24 | 20.13 | 21.38 | 21.75 | 20.63 | 0.69 | 0.3621 |
| Anion gap | 8–25 | 17.38 | 16.28 | 16.38 | 17.38 | 0.45 | 0.1527 |
| Serum cytokines and IgE |  |  |  |  |  |  |  |
| IL-6, pg/mL | --- | 79.28 | 17.78 | 89.58 | 65.34 | 0.66 | 0.5976 |
| TNF-α, pg/mL | --- | 228.77 | 54.66 | 56.72 | 151.40 | 0.35 | 0.1277 |
| IgE, ng/mL | --- | 179.70 | 178.69 | 121.21 | 254.69 | 63.06 | 0.5313 |
| ^1^University of Illinois Veterinary Medicine Diagnostics Laboratory Reference Ranges. | | | | | | | |

| **Supplementary Table 8.** Complete blood count and urine characteristics of healthy adult dogs after consuming test diets for 26 wk | | | | | | | |
| --- | --- | --- | --- | --- | --- | --- | --- |
| Item | Reference Range^1^ | Control | Low | Medium | High | SEM | *P*-value |
| Complete blood count |  |  |  |  |  |  |  |
| Red blood cells, 10^6^/µL | 5.50-8.50 | 7.28 | 7.22 | 6.96 | 7.52 | 0.19 | 0.2582 |
| Reticulocyte count, % | --- | 0.68 | 0.56 | 0.46 | 0.67 | 0.11 | 0.3915 |
| Hemoglobin, g/dL | 12.0-18.0 | 16.58 | 16.09 | 15.54 | 16.89 | 0.41 | 0.1322 |
| Hematocrit, % | 35.0-52.0 | 48.31 | 47.11 | 45.34 | 49.75 | 30.02 | 1.0000 |
| Mean cell volume, fl | 58.0-76.0 | 66.48 | 65.30 | 65.20 | 66.23 | 0.55 | 0.2640 |
| Mean corpuscular hemoglobin, pg | 20.0-25.0 | 22.75 | 22.29 | 22.34 | 22.46 | 0.25 | 0.5641 |
| Mean corpuscular hemoglobin, g/dL | 33.0-38.6 | 34.24 | 34.16 | 34.28 | 33.94 | 0.25 | 0.7725 |
| Platelets, 10^3^/µL | 200-700 | 301.50 | 356.87 | 280.25 | 322.25 | 37.19 | 0.5204 |
| White blood cell count, 10^3^/µL | 6.00-17.00 | 7.44 | 6.84 | 7.29 | 7.32 | 0.94 | 0.9874 |
| Lymphocytes, % | --- | 28.99 | 27.49 | 25.33 | 26.65 | 3.75 | 0.9553 |
| Monocytes, % | --- | 7.25 | 3.18 | 5.90 | 4.94 | 1.75 | 0.2605 |
| Eosinophils, % | --- | 2.35 | 2.46 | 3.93 | 2.91 | 0.48 | 0.1078 |
| Basophils, % | --- | 0.23 | 0.29 | 0.33 | 0.21 | 0.11 | 0.6397 |
| Lymphocytes, 10^3^/µL | 1.00-4.80 | 1.71 | 1.84 | 1.81 | 1.80 | 0.23 | 0.9316 |
| Monocytes, 10^3^/µL | 0.20-1.40 | 0.33 | 0.21 | 0.44 | 0.39 | 0.09 | 0.2925 |
| Eosinophils, 10^3^/µL | 0.10-1.00 | 0.16 | 0.17 | 0.27 | 0.21 | 0.04 | 0.2214 |
| Basophils, 10^3^/µL | 0.00-2.00 | 0.01 | 0.02 | 0.02 | 0.01 | 0.01 | 0.7289 |
| Urine characteristics |  |  |  |  |  |  |  |
| Specific gravity |  | 1.05 | 1.06 | 1.06 | 1.04 | 0.01 | 0.4598 |
| pH |  | 7.69 | 7.00 | 7.44 | 7.88 | 0.47 | 0.5912 |
| Total protein |  | 1.69 | 1.00 | 1.69 | 1.50 | 0.32 | 0.3847 |
| ^1^University of Illinois Veterinary Medicine Diagnostics Laboratory Reference Ranges. | | | | | | | |

**
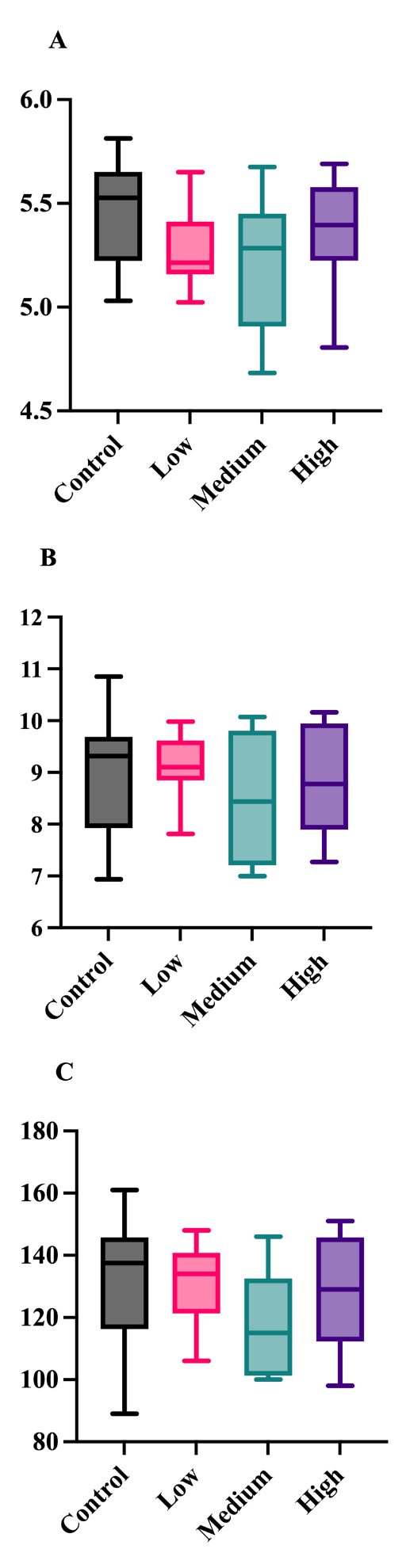
**

**Supplementary Figure 1.** Baseline fecal alpha diversity measures of healthy adult dogs after consuming the Control diet, including the Shannon Diversity Index (**A**; *P*=0.364), Faith’s phylogenetic diversity (**B**; *P*=0.873), and observed features (**C**; *P*=0.426).


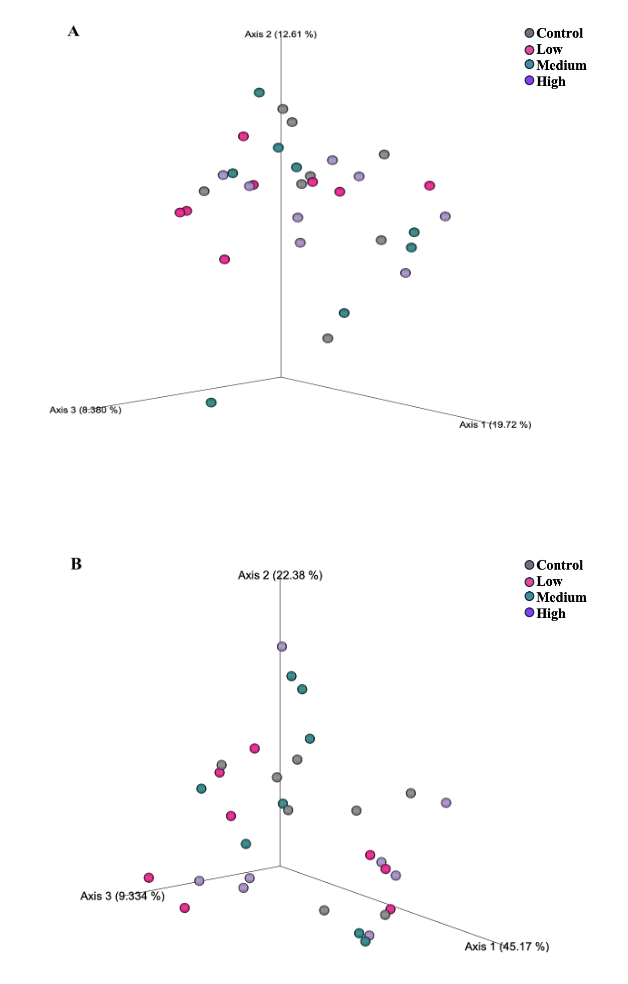


**Supplementary Figure 2.** Baseline fecal microbial communities of healthy adult dogs after consuming the Control diet, as represented as principal coordinates analysis plots of unweighted (**A**) UniFrac distances (*P*=0.767) and weighted (**B**) UniFrac distances (*P*=0.542). Each dot represents a sample collected from each dog (*n*=8/treatment).
